# Socioeconomic Differences in Cognitive Ability Across Childhood and Adolescence: An Investigation of Genetic, Individual, and Environmental Factors

**DOI:** 10.3390/jintelligence14040063

**Published:** 2026-04-10

**Authors:** Lena Paulus, Charlotte K. L. Dißelkamp, Andreas J. Forstner, Frank M. Spinath

**Affiliations:** 1 Department of Individual Differences & Psychodiagnostics, Saarland University, 66123 Saarbrücken, Germany; 2 Institute of Human Genetics, University Hospital Bonn, School of Medicine, University of Bonn, 53127 Bonn, Germany; 3 Institute of Neuroscience and Medicine (INM-1), Research Center Jülich, 52428 Jülich, Germany

**Keywords:** cognitive ability, socioeconomic status, intelligence development, parental cognitive ability, polygenic score

## Abstract

The level and development of cognitive ability are associated with parental socioeconomic status (SES). Some of these cognitive differences are presumably due to individual differences in genetic predispositions, but the potential mechanisms and influencing factors are still relatively unclear. Previous research has identified factors that show a relation with both cognitive abilities and SES (e.g., parental cognitive ability, home environment, and polygenic scores). Regarding these factors, we analysed three age cohorts (*N* = 6715; 5, 11, and 17 years old) at a 6-year interval using multiple regressions and decomposition analyses. Firstly, results indicated that cognitive differences linked to SES emerged particularly between the ages of 5 and 11. A substantial part of the SES effect was associated with parental cognitive ability. Secondly, particularly in the oldest cohort, the polygenic score for cognitive ability was related to the SES-associated change in cognitive ability. Finally, in several analyses, the influence of SES on cognitive ability was no longer significant after considering the attendance of the academic track in secondary school. This pattern could indicate that SES-associated differences in secondary school recommendations shown in previous studies may also be associated with SES-related differences in cognitive ability, which should be investigated in future studies.

## 1. Introduction

A child’s educational success depends on both their socioeconomic background (Harwell et al., 2017; OECD, 2022; Sirin, 2005) and their cognitive abilities (Deary et al., 2007; Roth et al., 2015). Differences in cognitive abilities can partly explain the socioeconomic status (SES) differences in education (Bukodi et al., 2014; Gil-Hernández, 2019; Paulus et al., 2021; von Stumm, 2017). The disparities in cognitive abilities between SES groups can already be observed at the very young age of 2 years and tend to increase with age (Feinstein, 2003; von Stumm & Plomin, 2015). However, no conclusive explanation for this disparity has been reported yet. In part, these differences may be linked to measurable SES-related factors that impact cognitive development, as has already been shown for the factor “reading to the child” (Sullivan & Brown, 2013). In addition, differences in genetic predisposition might also be a contributing factor to the SES gap (Hanscombe et al., 2012). Therefore, this study was designed to investigate the effect of genetic, individual, and environmental factors on SES differences in cognitive ability and the change in cognitive ability across three different age cohorts.

### 1.1. Development of Cognitive Abilities and the Link to SES

Individuals with higher cognitive abilities are more likely to complete more years of education, achieve a higher income, and have a higher life expectancy (Deary et al., 2008; Strenze, 2007). In general, cognitive abilities show modest stability in the preschool age, medium stability in childhood, and high stability from adolescence to adulthood (Breit et al., 2024).

Cognitive abilities and their development can be influenced by a number of factors, e.g., schooling (Judd et al., 2022) or reading to the child (Sullivan & Brown, 2013). One major factor that is associated with the cognitive abilities of a child as well as their development is parental SES (Judd et al., 2022; Korous et al., 2022; Strenze, 2007; von Stumm & Plomin, 2015). As early as 2 years of age, children from high SES families display higher cognitive abilities compared to children from low SES families, including, for example, language skills, vocabulary, executive functioning, and memory (Feinstein, 2003; Maaz et al., 2022; Noble et al., 2015; Romeo et al., 2022; von Stumm & Plomin, 2015). Overall, the difference in cognitive abilities between children from differing SES backgrounds appears to be relatively constant at preschool age (i.e., from 3 to 5 years of age; Feinstein, 2003; Maaz et al., 2022; Romeo et al., 2022), but previous research suggests that the disparities in cognitive abilities continue to increase during the primary school years and up to the age of 16, as SES effects appear to accumulate (Feinstein, 2003; von Stumm & Plomin, 2015).

High cognitive ability contributes to a higher level of education and occupation as well as a higher income (Strenze, 2007). Thus, parents who have achieved a good education and a high income, and therefore a higher SES, are also more likely to show a higher cognitive ability than parents with a poorer education or lower income. Since cognitive ability is partly genetic (Haworth et al., 2010; Tan et al., 2025), the cognitive abilities of parents and children are linked (Eriksen et al., 2013; Tong et al., 2007), which could explain part of the SES effect. However, SES is still associated with cognitive ability even when controlling for maternal cognitive ability (Eriksen et al., 2013; Tong et al., 2007). In addition, SES effects have been found in adoption studies (Kendler et al., 2015), indicating that parental SES must also be related to children’s cognitive abilities through further non-genetic pathways. It is possible that high SES children already show better language development before starting school and therefore have more resources available for developing their skills. This reasoning is supported by the finding that SES differences in executive functioning can be explained by advantages in the early language development of children with high SES (Romeo et al., 2022). However, it could also be the case that due to more available resources or greater familiarity with higher education and the education system, high SES parents can support their children more effectively or create a better learning environment compared to low SES parents, which in turn may lead to a widening of the cognitive abilities gap (Aschaffenburg & Maas, 1997; Boonk et al., 2018; Bradley & Corwyn, 2002; Sullivan, 2001). Finally, high SES children’s cognitive abilities could develop more positively than those of children with a low social status, as social status may be associated with further variables that promote cognitive development. In this context, gene–environment interplay could also be a pivotal factor, as certain genetic traits may be supported differently depending on the SES of a family or the genetic traits could be exposed to different environments, which in turn could affect cognitive abilities (Sauce & Matzel, 2018). One factor that has already been found to explain part of the SES effect on children’s cognitive abilities is reading to the child (Sullivan & Brown, 2013). Further factors, which have been shown to be related to cognitive abilities as well as parental SES, such as openness or home environment (Anglim et al., 2022; Ayoub et al., 2018; Hart et al., 2007), could therefore also be relevant for understanding SES-related differences in cognitive abilities. In the following section, both individual and environmental influencing factors that have been shown in previous research to be associated with parental SES as well as cognitive ability, and could therefore may help explain the SES disparities in cognitive ability, are presented.

### 1.2. Individual Traits and Their Influence on Cognitive Abilities

In previous research, several individual traits have been shown to affect the cognitive abilities of children, such as self-control (Meldrum et al., 2017) or a child’s personality (Anglim et al., 2022; Lechner et al., 2017). In particular, a child’s openness to experience, one of the personality factors in the five-factor model (McCrae & Costa, 1987), could play a role in the SES disparities, as it appears to be related to both cognitive abilities (Anglim et al., 2022; Lechner et al., 2017) and parental SES (Ayoub et al., 2018). Openness is also a significant predictor of academic achievement, albeit to a lesser extent than for cognitive abilities (Bergold & Steinmayr, 2018; Laidra et al., 2007; Mammadov, 2022; Marsh et al., 2020; Meyer et al., 2019, 2023; Poropat, 2009). The link between openness and crystallised intelligence is stronger than for fluid intelligence (Anglim et al., 2022), and openness shows a larger effect for standardised test achievement than for the more subjective school grades (Lechner et al., 2017; Meyer et al., 2019; Spengler et al., 2013). Moreover, openness has been found to play a key role in social mobility: higher openness leads to higher cognitive abilities and education even after controlling for childhood SES, which then indirectly leads to a higher SES as an adult at age 64 (Staff et al., 2017). In addition, childhood cognitive abilities—as well as SES and education—in turn affect adult openness (Furnham & Cheng, 2016).

Furthermore, children’s cognitive abilities are influenced to a significant extent by genetics, which has already been shown in quantitative genetic studies, often utilising a twin design (Hanscombe et al., 2012; Haworth et al., 2010; Spinath et al., 2003). Overall, these studies indicate that the genetic influence grows larger with age (Haworth et al., 2010), which could in part be due to a form of gene–environment correlation (r_GE_). The expression of cognitive abilities and other traits may also be affected by the interplay of genes and the environment, which is called gene–environment interaction (GxE). Whereas GxE implies that certain environments can have different effects depending on the genotype, r_GE_ entails that individuals with a certain genotype experience certain environments more frequently. As these experiences influence future cognitive development, the effects of r_GE_ can accumulate over the course of life and could in turn explain why the importance of genetic effects increases with age (Sauce & Matzel, 2018).

An alternate approach to analysing genetic influence originates in molecular genetic research. In recent years, polygenic scores (PGS) have been increasingly used as indicators for genetic influence, which are derived from genome-wide association studies (GWAS). These PGS reflect aggregated scores for each individual which are based on the presence of a large number of genetic variants (single nucleotide polymorphisms; SNPs) across the genome. Thus, PGS are predictive indicators that can be used at the population level to analyse genetic influences. At present, only part of the genetic influence and total variance reported in quantitative genetic studies can be explained by PGS, namely 7–10% of the variance in cognitive ability (Lee et al., 2018). In a within-family GWAS design, which, unlike other GWAS, controls for environmental confounding and indirect genetic effects such as population stratification, assortative mating, or genetic nurture, the direct genetic effect on cognitive ability is estimated at 14–18.8% (Howe et al., 2022; Tan et al., 2025). The association of a PGS for cognitive abilities (PGS_CA_) with cognitive abilities and educational achievement has already been replicated in different cohorts ranging from children to adults (Allegrini et al., 2020; Demange et al., 2021; Genç et al., 2021; Gorelik et al., 2024; Loughnan et al., 2023). Children with higher PGS_CA_ also tend to grow up in families with higher parental SES^1^ (Demange et al., 2021; Gorelik et al., 2024; Judd et al., 2022), which could jointly shape cognitive abilities. By including PGS_CA_, it can be examined to what extent genetic differences contribute to the explanation of the SES effect on cognitive abilities, which could indicate gene–environment correlation (r_GE_) as a possible mechanism. PGS_CA_ still shows an independent effect on children’s cognitive abilities even when SES is taken into account (Judd et al., 2022), which could indicate an independent genetic contribution.

Since cognitive abilities have this genetic component (Haworth et al., 2010), the cognitive abilities of children and the cognitive abilities of parents are correlated beyond what can be explained by shared environmental factors (Anger & Heineck, 2010; Eriksen et al., 2013; Tong et al., 2007). Even after controlling for SES, maternal or parental cognitive abilities contribute significantly to the child’s cognitive development (Eriksen et al., 2013; Ronfani et al., 2015; Schulz et al., 2017; Tong et al., 2007; Torres, 2013), with maternal cognitive ability appearing to contribute in part to SES differences in cognition (Marks & O’Connell, 2023). Overall, maternal cognitive abilities appear to be more important for the transmission of cognitive abilities than those of the father (Anger & Heineck, 2010). In conclusion, Eriksen et al. (2013) suggest that parental cognitive abilities—as well as parental education—should be included in analyses investigating the effects of other predictors of cognitive abilities in order to avoid spurious associations.

### 1.3. Environmental Influences on Cognitive Abilities and Educational Attainment

In addition to genetic influences, environmental factors can also affect the development of cognitive abilities and later educational attainment. However, it is important to emphasise that these two influencing factors cannot be clearly disentangled: PGS in part reflect environmental aspects (Sauce & Matzel, 2018; Tan et al., 2025), and environmental variables, e.g., home environment, can also be influenced by genetics (Starr & Riemann, 2022). The fact that the environment plays a decisive role in the development of cognitive abilities is shown by two findings: (1) when children are adopted and grow up in families with high SES, their cognitive ability scores increase in contrast to their siblings who remained in the original family (Kendler et al., 2015); and (2) when children with low SES improve their cognitive ability scores through intervention programmes, this gain disappears after the end of the intervention (Protzko, 2015). Those environmental factors that ultimately lead to differences in the development of cognitive abilities in children with differing SES levels are still largely undetermined. However, some factors that show a positive association with cognitive abilities and their development have been studied and will be introduced in the following section.

Besides the cognitive abilities of parents, parental behaviours, e.g., reading to children, can impact the cognitive abilities of children. Reading to one’s child seems to improve reading ability, language skills, and cognitive abilities (McGinnity et al., 2022; Schneider & Linberg, 2022; Sullivan & Brown, 2013), with high SES parents generally reading to their children more frequently than low SES parents (McGinnity et al., 2022). Frequent reading to children at the age of 5 appears to have a positive effect on cognitive abilities at age 16, even when parental education is taken into account (Sullivan & Brown, 2013). Reading to the child even explains part of the effect that parental education exerts on the cognitive abilities of adolescents (Sullivan & Brown, 2013). Similarly, for vocabulary development and reading ability, reading to the child has been shown to be an influencing factor in explaining SES differences (Maaz et al., 2022; McGinnity et al., 2022; Schneider & Linberg, 2022).

Children who grow up in a household with a high quality of home environment also show higher levels of cognitive abilities and are more likely to live with parents of higher SES (Deater-Deckard et al., 2009; Hart et al., 2007; Matheny et al., 1995; Petrill et al., 2004). A poor-quality home environment is defined by a high level of subjectively perceived noise, crowding, and traffic within the home (Matheny et al., 1995). The quality of home environment is also an important factor for related constructs, including executive functioning and receptive vocabulary (Berry et al., 2016), expressive vocabulary and phonological awareness skills (Johnson et al., 2008), as well as educational achievement (Allegrini et al., 2020; Garrett-Peters et al., 2016; Hanscombe et al., 2011; Marsh et al., 2020). Moreover, the home environment still shows an effect on educational achievement even when controlling for both PGS and SES (Cheesman et al., 2022).

In sum, a number of individual and environmental factors have already been identified that can influence children’s cognitive abilities. However, these have rarely been analysed jointly or studied in relation to the development of cognitive abilities across different SES backgrounds.

### 1.4. The Aim of the Present Study

A child’s cognitive development appears to be associated with their SES (Feinstein, 2003; von Stumm & Plomin, 2015). Why exactly cognitive development could differ across SES backgrounds, however, has not yet been fully understood. This study therefore aimed to analyse the effects of SES, parental cognitive ability, individual (openness and PGS_CA_), and environmental factors (reading to the child and home environment) on children’s cognitive ability and the change in cognitive ability in order to identify the factors linked to the cognition gap between children from differing SES backgrounds.

In addition to administering the same cognitive ability test at both time points with an average measurement interval of 6 years, three different age cohorts (5, 11, and 17 years old) ranging from early childhood to young adulthood were analysed. This allowed the change in cognitive ability to be examined over a longer period of time and furthermore allowed us to determine whether the effect of the predictors varied across different stages of development (Tong et al., 2007). For example, it can be assumed that environmental variables, such as the quality of home environment, do not impact the change in cognitive ability in the oldest cohort, or at least to a much lesser extent. Moreover, the stability of cognitive ability increases with age (Breit et al., 2024), suggesting that changes in cognitive ability could be smaller with increasing age of the cohorts. Patterns of SES-related differences at an age older than 16 seem to have been barely considered to date.

The following hypotheses were tested:

(1) A higher SES is associated with higher (a) cognitive ability and (b) gain in cognitive ability than a lower SES
(2) PGS_CA_, openness, home environment, parental cognitive ability, and reading to the child can statistically account for part of the variance in (a) cognitive ability and (b) change in cognitive ability, with higher levels of the predictors expected to be associated with better outcomes
(3) PGS_CA_, openness, home environment, parental cognitive ability, and reading to the child can statistically account for SES-related differences in (a) cognitive ability and (b) change in cognitive ability.

## 2. Methods

### 2.1. Sample

For the present study, all cohorts except for the oldest cohort of the longitudinal TwinLife study were analysed (Diewald et al., 2024). At the time of the first assessment, the twins were around 5 (cohort 1), 11 (cohort 2), and 17 (cohort 3) years old. In TwinLife, not only were data on monozygotic and dizygotic same-sex twin pairs collected, but also on siblings and parents. Overall, in the first assessment, 4096 families participated, which were recruited using a probability-based sampling design. Since 2014, there have been a total of 11 surveys over a period of 10 years. In the TwinLife study, higher-educated households are slightly overrepresented. Nevertheless, the sample is suitable for analyses of social inequality, as it covers the entire distribution of socio-demographic indicators (e.g., educational status, occupational status, and income) (for further information, see Hahn et al., 2016; Lang & Kottwitz, 2017; Rohm et al., 2023). The TwinLife study was ethically approved by the German Psychological Association (Deutsche Gesellschaft für Psychologie; protocol number: RR 11.2009) and complied with the ethical standards of the 1964 Declaration of Helsinki and its subsequent amendments. Consent of the participants was obtained through verbal and, for the genetic data, written informed consent. Collection and use of the molecular genetic data was ethically approved by the ethics committees of the University of Bielefeld, Germany (EUB 2015-043). Data from 6226 twins in three age cohorts as well as 489 siblings within the same age range were used for the present study. The inclusion criterion for the present study was the participation in the first wave of the TwinLife survey.

### 2.2. Measurements

#### 2.2.1. Parental Socioeconomic Status (SES)

Education, income, occupation, and occupational class of both parents were taken into account for the calculation of parental socioeconomic status (Bukodi et al., 2014). To this end, the International Standard Classification of Education (ISCED; UNESCO, 2011) was used as a measure of a mother’s and father’s education, the International Socioeconomic Index of Occupational Status (ISEI; Ganzeboom et al., 1992) as a measure of their occupational status, the Erikson-Goldthorpe-Portocarero Class Schedule (EGP; Erikson et al., 1979) as an indicator of class, and lastly, household income, adjusted using the modified OECD scale (OECD, 2013). If the monthly household income from the first face-to-face interview (F2F 1) appeared implausible, i.e., very low (<1000 €) or very high (>20,000 €), it was checked against the gross income and longitudinally. If the value was confirmed as implausible, it was set to missing (*n* = 112) or, if possible and plausible, the information from the second face-to-face interview (F2F 2) was taken into account (*n* = 107). In addition, if the value from F2F 1 was not available, the checked value from F2F 2 was included (*n* = 205). The income variable was log-transformed. The total SES value was calculated in a latent factor analysis in Mplus (Version 8.2; Muthén & Muthén, 2017), which allowed the ordinal scaling of EGP and ISCED to be taken into account. SES was then residualised for parental age. Since SES appears to be stable over time (Hanscombe et al., 2011), we included the measurements from the first assessment (*n* = 6702). Cronbach’s α for all scales is reported in Table 1.

#### 2.2.2. Cognitive Ability

**Figure 1:**
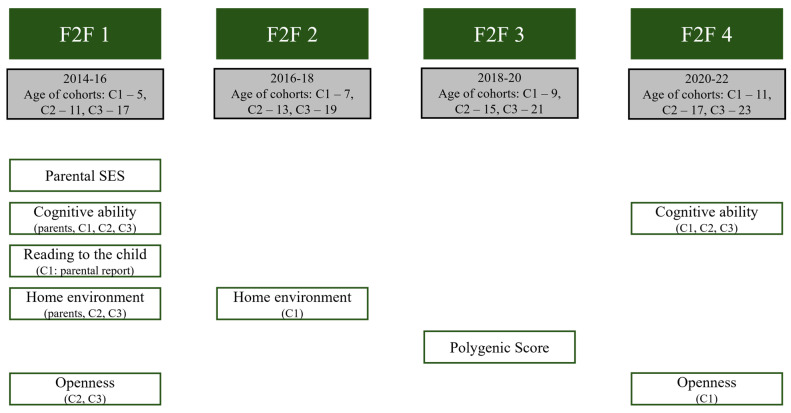
Constructs and measurement time points (F2F = face-to-face interview, C1 = cohort 1, C2 = cohort 2, and C3 = cohort 3).

Cognitive ability was tested using the non-verbal ‘Grundintelligenztest Skala 2′ (CFT 20-R [Culture Fair Intelligence Test], Revision; Weiß, 2006) according to Cattell’s concept of fluid intelligence (Gruber & Tausch, 2016). The test was applied both at the first measurement point (F2F 1: 2014–2016) and at the fourth household interview (F2F 4: 2020–2022), so that there was an average of 6.03 years between the two measurement points. An overview of the survey dates for all variables can be found in Figure 1. The CFT 20-R consisted of the four subtests ‘Figural Reasoning’ (15 items), ‘Figural Classification’ (15 items), ‘Matrices’ (15 items), and ‘Reasoning’ (11 items) with a total of 56 items. The youngest cohort 1 (=C1) forms an exception. As the twins and siblings in this age range were still too young for the CFT 20-R at the age of 5 at the time of the first interview, the CFT 1-R (Weiß & Osterland, 2013) was used instead. The CFT 1-R consisted of a total of 45 items with the subtests ‘Figural Reasoning’ (15 items), ‘Figural Classification’ (15 items), and ‘Matrices’ (15 items), which deviated from the CFT 20-R in terms of item difficulty. The subtests were combined into a cognitive ability factor using factor analysis, separately for each cohort and measurement time point. Parents’ cognitive abilities were assessed only at the first measurement point and were included separately for each parent, whereby they were residualised for maternal and paternal age.

#### 2.2.3. Polygenic Score (PGS_CA_)

We computed a polygenic score using existing genotype data from the TwinSNPs study, a molecular genetic subproject of the TwinLife study. DNA was extracted from saliva samples collected during multiple waves: 2018–2020 (“F2F 3”), 2021–2022 (“Cov 3”), and 2022–2024 (“F2F 5”). Genotype data preprocessing and polygenic score computation were conducted for the full genotyped TwinSNPs sample. The analysis sample used in the present study constitutes a subset of this larger TwinSNPs cohort. Further details on genotype quality control, imputation, and polygenic score calculation are provided in Supplement B-I of Deppe et al. (2026). The PGS_CA_ used in the present study is based on a large-scale genome-wide association study (GWAS; *N* = 257,841; Lee et al., 2018) of cognitive test performance and was computed using the PRS-CS auto model (Ge et al., 2019). To ensure compatibility with the discovery GWAS, we restricted the analysis to participants of genetically inferred European ancestry. We further excluded individuals flagged as population outliers or whose genetically inferred sex or family assignment did not match self-reported data. Before analysis, the PGS_CA_ was residualised for the first ten principal components of genetic ancestry to account for population stratification (*N* = 2535).

#### 2.2.4. Home Environment

The quality of home environment was measured using the “Confusion, Hubbub and Order (CHAOS) Scale” (Matheny et al., 1995). This questionnaire consists of a total of six items (“I have a regular bedtime routine”, “You can’t hear yourself think at home”, “It’s a real zoo at home”, “We are usually able to keep track of things”, “There is usually a TV on somewhere at home”, and “The atmosphere in our house is calm”), which were answered on a five-point Likert scale ranging from “strongly disagree” to “strongly agree”. The questionnaire was answered by both the children and the parents. For parents and cohorts 2 and 3, the questions were collected in F2F 1; cohort 1 answered the questions in F2F 2. For the twins and siblings who no longer lived with their parents, the questions were asked retrospectively. A total score was calculated separately for the parents and the child. Since a single-factor aggregation proved to be as good a fit as a two-factor aggregation (e.g., CFI = 0.961 vs. CFI = 0.963), the single-factor solution was considered in this study. Since the oldest cohort did not answer item 1 (“I have/had a regular bedtime routine”), this item was excluded from all cohorts and parents to ensure comparability. In addition, this exclusion also resulted in better fit indices (e.g., CFI = 0.986). If the score was available for both parents (60.4% of cases), their score from the two latent factor analyses was averaged. If not, only the score of the parent with available data was included in the analysis.

#### 2.2.5. Openness

In TwinLife, personality was assessed using the Big Five Inventory—Short Version (BFI-S; Gerlitz & Schupp, 2005) with a total of 15 items. Openness to experience was measured using three items (“I see myself as someone who…” “is original, comes up with new ideas”, “values artistic, aesthetic experiences”, “has an active imagination”). In a comparative study, the BFI-S showed good reliability (Gerlitz & Schupp, 2005). For cohorts 2 and 3, openness was assessed in F2F 1, for cohort 1 in F2F 4. Consequently, openness for cohort 1 was not included in the cross-sectional analysis.

#### 2.2.6. Reading to the Child

In F2F 1, one question from the German Family Panel pairfam was adapted to survey reading to the child (Pairfam Group, 2022): “How often did you or your partner or someone else in the family take part in one of the following activities with [name of the child] in the last four weeks?”—“Reading books or stories aloud”. The response options were “not at all”, “about once a month”, “about once a week”, “several times a week”, and “(almost) daily”. Due to the advanced age of the other cohorts, this variable was only recorded for cohort 1, who were 5 years old at the time of the survey. The ordinal variable was averaged over the parents. After checking that the results did not deviate when considering the ordinal variable as interval-scaled, the variable was included in the analysis as an interval-scaled variable to simplify the analyses.

#### 2.2.7. Control Variables

In a separate step, as control variables, we included the age and sex of the children as well as years of schooling and attendance at the academically orientated school (=“Gymnasium”), since more years of schooling and attending the academic track in secondary school are associated with improvement in cognitive ability (Becker et al., 2012; Judd et al., 2022; Ritchie & Tucker-Drob, 2018; Skopek & Passaretta, 2021). Schooling itself does not appear to contribute to either narrowing or widening the cognition gap that pre-exists due to SES or genetic factors, so that all children seem to profit from schooling equally (Judd et al., 2022; Skopek & Passaretta, 2021). However, since children with a higher SES are more likely to attend a higher-level secondary school than children with a lower SES even when they perform equally well in cognitive or achievement tests (Arnold et al., 2007; Gil-Hernández, 2019), this, too, may affect cognitive development associated with SES. In addition, the birth weight of the children was also considered as a control variable (lower/higher than 2500 g; Kormos et al., 2014).

### 2.3. Analyses

For the outcomes (1) cognitive ability at the first time point and (2) change in cognitive ability, separate multiple regression analyses for each cohort were computed, including parental SES, openness, maternal cognitive ability, paternal cognitive ability, home environment, PGS_CA_, reading to the child (cohort 1 only), and the control variables age, sex, and birth weight to test for the main effect of these predictors. For the outcome change in cognitive ability, it was tested whether the predictors showed an incremental effect when the assessment of cognitive ability at the first time point (F2F 1) was included in the analyses. A residual change model was applied, as only two measurement points of cognitive data were available. One aim of these analyses was to determine whether SES is related to variation in cognitive ability in all cohorts at both time points and whether SES-related differences in cognitive ability increase over time and age. In the next step, multiple mediation analyses were conducted, separately for each outcome and cohort^2^. Again, the control variables sex, age, and birth weight, as well as cognitive ability at time point 1, were taken into account in the analyses. Mediation analyses were applied to decompose the differences in cognition associated with SES into direct and indirect pathways (=decomposition analyses). Given the interrelationship of several predictors with SES through common background processes (e.g., parental cognitive abilities, PGS_CA_), the results were interpreted as associations rather than causal mechanisms. The analyses were conducted both cross-sectionally and longitudinally. We performed additional analyses for years of schooling and attendance of the academic track in a last step in all analyses. Years of schooling and attendance of the academic track were also considered as exploratory indirect pathways (Supplementary Tables S6 and S7). In all analyses, the family-level clustering of the data was taken into account^3^. The data were processed in IBM SPSS Statistics (Version 29; IBM Corp., 2024) and analyses were conducted using Mplus (Version 8.2; Muthén & Muthén, 2017). To calculate an overall score for the predictors SES, cognitive ability, home environment, and openness, latent factor analyses were conducted, separately for each cohort. All predictors, except for reading to the child, were mean-centred. In addition to these analyses, the PGS_CA_ of the parents was controlled in a non-pre-registered and thus exploratory analysis to disentangle if a direct PGS effect rather reflected a genetic or environmental influence (Tan et al., 2025; Supplementary Tables S8 and S9). To compare SES effects to previous research, we also conducted analyses without parental cognitive ability (Supplementary Tables S4 and S5).

As in other panel studies, the participation rate in TwinLife decreased with each survey, especially after the first survey wave (see Rohm et al., 2023 for more details). Dropout up to F2F 4 was not random but was related to a higher cognitive ability score in F2F 1 and a higher SES (e.g., mother’s education). These discrepancies were determined separately for each cohort and included as auxiliary variables in the estimation of missing values. To account for missing data, full information maximum likelihood (FIML) estimation was applied (for robustness analyses, see Supplementary Tables S10 and S11). To take the dropout into account when analysing the correlations, these were also calculated in Mplus.

### 2.4. Transparency and Openness

We report how we determined our sample size, all data exclusions, all manipulations, and all measures in the study. The study complies with the reporting standards for quantitative research in psychology (Appelbaum et al., 2018). The analysis code is available in the Supplementary Material (Syntax 1–2). All data and research materials can be accessed via Gesis. The TwinLife data (Diewald et al., 2024) is available for research purposes free of charge after signing a data use agreement^4^. The hypotheses and the research and analysis plan were pre-registered in the Open Science framework after data collection but before analyses were carried out [https://osf.io/2hfja (accessed on 20 February 2025)].

## 3. Results

### 3.1. Descriptive Statistics and Correlations

**Figure 2:**
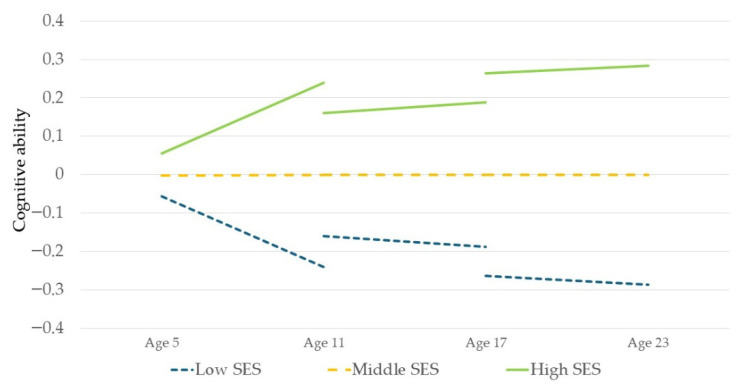
Cohort comparison and longitudinal change in the level of cognitive ability at both time points, calculated by separate multiple regression (separated by time point and cohort) with only the predictor parental SES. Low/high SES = deviation of minus or plus 1 standard deviation in the regression. For better comparability of the time points, only the complete sample was considered (*n* = 765, 772, and 704 per cohort).

Descriptive statistics and correlations are depicted in Table 1 and Table 2. As expected, the CFT sum values increased with age. The correlation of the two measurement points for cognitive ability was higher in the oldest cohorts than in the younger cohort. Apart from home environment, all included variables were significantly correlated with both parental SES and cognitive ability across all cohorts. When the cohorts were of the same age (e.g., cohort 1 at F2F 4 and cohort 2 at F2F 1), the correlations between cognitive ability and SES, or the cognitive ability of parents and children, were comparable in magnitude. In addition, regression analyses were performed using only the SES predictor to depict the comparability of the cohorts (Figure 2).

### 3.2. Cross-Sectional Multiple Regression

In the multiple regression with the outcome cognitive ability at the first time point (F2F 1) (Table 3), maternal cognitive ability (C1: β = 0.15; *p* < .001; *SE* = 0.03; C2: β = 0.20; *p* < .001; *SE* = 0.03; C3: β = 0.20; *p* < .001; *SE* = 0.03), paternal cognitive ability (C1: β = 0.10; *p* = .024; *SE* = 0.05; C2: β = 0.21; *p* < .001; *SE* = 0.03; C3: β = 0.24; *p* < .001; *SE* = 0.03), and parental home environment (C1: β = 0.07; *p* = .013; *SE* = 0.03; C2: β = 0.07; *p* = .004; *SE* = 0.02; C3: β = 0.06; *p* = .015; *SE* = 0.03) were associated with cognitive ability across cohorts. The predictor PGS_CA_ (C2: β = 0.10; *p* = .007; *SE* = 0.04; C3: β = 0.10; *p* = .029; *SE* = 0.05) showed a significant effect in the two older cohorts, but not in cohort 1. After controlling for the attendance of the academic track (C2: β = 0.26; *p* < .001; *SE* = 0.02; C3: β = 0.22; *p* < .001; *SE* = 0.02), SES was no longer significantly related to variation in cognitive ability. In addition, for cohort 3, openness showed a significant effect (β = 0.06; *p* = .005; *SE* = 0.02). The variance explained (*R*^2^ = 9.7%; *p* < .001; *SE* = 0.02) was significantly lower in the youngest cohort than in the two older cohorts (C2: *R*^2^ = 30.0%; *p* < .001; *SE* = 0.02; C3: *R*^2^ = 31.0%; *p* < .001; *SE* = 0.02). The multiple regression and decomposition models were saturated, so that no model fit was available.

### 3.3. Longitudinal Multiple Regression

For the change in cognitive ability (model 2, Table 4), which was analysed by controlling for cognitive ability at the first measurement point (F2F 1), the pattern of the contributing predictors differed between the cohorts. For cohort 1, SES (β = 0.12; *p* = .034; *SE* = 0.06), maternal cognitive ability (β = 0.12; *p* = .010; *SE* = 0.05), paternal cognitive ability (β = 0.23; *p* < .001; *SE* = 0.06), openness (β = 0.10; *p* = .006; *SE* = 0.04), and cognitive ability in F2F 1 (β = 0.13; *p* = .001; *SE* = 0.04) were significantly associated with the outcome change in cognitive ability between the ages of 5 and 11. For cohort 2, the significant predictors were maternal cognitive ability (β = 0.15; *p* < .001; *SE* = 0.04), birth weight (β = −0.06; *p* = .033; *SE* = 0.03), and cognitive ability in F2F 1 (β = 0.53; *p* < .001; *SE* = 0.03), and for cohort 3, PGS_CA_ (β = 0.12; *p* = .002; *SE* = 0.04), sex (β = −0.13; *p* < .001; *SE* = 0.03), and cognitive ability in F2F 1 (β = 0.61; *p* < .001; *SE* = 0.03). With the exception of SES in cohort 1 (β = 0.07; *p* = .188; *SE* = 0.06), these effects were stable when controlling for the attendance of the academic track, which was also significantly associated with the change in cognitive abilities across all cohorts (C1: β = 0.14; *p* = .002; *SE* = 0.04; C2: β = 0.16; *p* < .001; *SE* = 0.04; C3: β = 0.09; *p* = .016; *SE* = 0.04). For the outcome cognitive ability in F2F 4, the results were mostly comparable to the cross-sectional analysis, as the significant predictors were SES (not for C2, but for C1: β = 0.11; *p* = .049; *SE* = 0.06 and C3: β = 0.10; *p* = .019; *SE* = 0.04), maternal cognitive ability (C1: β = 0.14; *p* = .002; *SE* = 0.05; C2: β = 0.28; *p* < .001; *SE* = 0.04; C3: β = 0.17; *p* < .001; *SE* = 0.04), paternal cognitive ability (C1: β = 0.25; *p* < .001; *SE* = 0.06; C2: β = 0.22; *p* < .001; *SE* = 0.05; C3: β = 0.22; *p* < .001; *SE* = 0.05), and PGS_CA_ (C3: β = 0.21; *p* < .001; *SE* = 0.05). In addition, openness explained variance not only in the cohort 3 model (β = 0.07; *p* = .039; *SE* = 0.03), but also in cohort 1 (β = 0.10; *p* = .006; *SE* = 0.04). In contrast, for F2F 4, the parental home environment only showed an effect for cohort 3 (β = 0.09; *p* = .031; *SE* = 0.04). The variance explained by the models was between 21.1% (*p* < .001; *SE* = 0.04; C1: model 1) and 56.9% (*p* < .001; *SE* = 0.03; C3: model 3).

### 3.4. Cross-Sectional Decomposition Analysis

In order to examine which predictors were associated with the SES-related differences in cognitive ability, decomposition analyses were conducted (Table 5). For all cohorts, significant indirect effects were found for maternal cognitive ability (C1: β = 0.08; *p* < .001; *SE* = 0.02; C2: β = 0.10; *p* < .001; *SE* = 0.01; C3: β = 0.09; *p* < .001; *SE* = 0.01) and paternal cognitive ability (C1: β = 0.05; *p* = .024; *SE* = 0.02; C2: β = 0.11; *p* < .001; *SE* = 0.02; C3: β = 0.11; *p* < .001; *SE* = 0.02). In addition, significant indirect effects were found for PGS_CA_ (β = 0.02; *p* = .030; *SE* = 0.01) in cohort 2, and for parental home environment (β = 0.01; *p* = .022; *SE* = 0.01) and openness (β = 0.01; *p* = .026; *SE* = 0.00) in cohort 3. In an exploratory analysis, the attendance of the academic track was also associated with part of the SES effect in both older cohorts (Supplementary Table S6; C2: β = 0.11; *p* < .001; *SE* = 0.01; C3: β = 0.08; *p* < .001; *SE* = 0.01).

### 3.5. Longitudinal Decomposition Analysis

For the outcome change in cognitive ability, decomposition analyses were conducted, separately for each cohort and controlling for the first measurement of cognitive ability (Table 6; for all models, see Supplementary Tables S1–S3). In cohort 1, significant indirect effects were found for cognitive ability in F2F 1 (β = 0.01; *p* = .017; *SE* = 0.01), maternal cognitive ability (β = 0.06; *p* = .018; *SE* = 0.02), paternal cognitive ability (β = 0.11; *p* < .001; *SE* = 0.03), and openness (β = 0.02; *p* = .026; *SE* = 0.01). For cohort 2, significant indirect effects were found for cognitive ability in F2F 1 (β = 0.16; *p* < .001; *SE* = 0.02), maternal cognitive ability (β = 0.07; *p* < .001; *SE* = 0.02), and paternal cognitive ability (β = 0.05; *p* = .046; *SE* = 0.03); and for cohort 3, cognitive ability in F2F 1 (β = 0.20; *p* < .001; *SE* = 0.02), and PGS_CA_ (β = 0.02; *p* = .021; *SE* = 0.01). In an exploratory analysis, the track attendance and years of education were examined as potential indirect pathways (Supplementary Table S7), with track attendance showing a significant indirect effect on the SES-related change in cognitive ability in all cohorts (C1: β = 0.06; *p* = .002; *SE* = 0.02; C2: β = 0.07; *p* < .001; *SE* = 0.02; C3: β = 0.03; *p* = .017; *SE* = 0.01).

## 4. Discussion

In this study, cognitive ability as well as the change in cognitive ability were analysed in relation to parental SES and further individual and environmental predictors, both cross-sectionally and longitudinally across three different German age cohorts. By including three different age cohorts, this study contributes to extending knowledge on the emergence and developmental timing of the SES-related cognitive gap from early childhood to early adulthood. The results provide initial evidence of which predictors may play a role in the emergence of SES differences in cognition. In addition, these findings suggest that the relevance of certain factors—such as parental cognitive abilities and PGS_CA_—may vary across different stages of development. The results showed that higher parental SES was associated with higher levels of cognitive ability and the change in cognitive ability. However, when additional predictors were included in the model, especially parental cognitive ability, the effect of SES was greatly reduced or no longer significant.

In the youngest cohort, at the age of 5, maternal cognitive ability was particularly associated with a child’s level of cognitive ability, with the home environment as reported by the parents also showing a small association. The SES effect initially appeared to be rather small (*r* = 0.10) and statistically overlapped with the cognitive abilities of parents, which were also strongly associated with changes in cognitive ability up to the age of 11. The cognitive abilities of the mother, openness and academic track attendance, were likewise related to the change in cognitive ability. In the second oldest cohort, which covered the age range from 11 to 17 years, parental cognitive abilities again were substantially related to cognitive ability at the age of 11. In addition, the home environment as reported by the parents, PGS_CA_ and academic track attendance were linked to a child’s cognitive ability. Attendance of the academic track could also be associated with part of the SES differences, as SES was no longer significant after including academic track attendance. Here, too, the SES effect was primarily related to parental cognitive ability and, in addition, PGS_CA_. Cognitive performance at age 11 predicted the change in cognitive ability at age 17. However, parental cognitive abilities continued to be associated with the child’s cognitive ability. The SES differences appeared to increase only slightly across this age range. A large part of the SES difference reflected pre-existing differences at the age of 11, such that children with higher SES already showed higher cognitive performance than children with lower SES, which persisted at the age of 17. In addition, maternal cognitive ability was also linked to the SES-associated differences at age 17. Even in the oldest cohort, between the ages of 17 and 23, the SES disparities appeared to increase slightly. At the age of 17, parental cognitive abilities, parent-reported home environment, PGS_CA_, openness, and attendance of the academic track were related to children’s cognitive abilities. As in cohort 2, academic track attendance appeared to be associated with the SES differences. In the decomposition analysis, the SES differences were linked to parental cognitive abilities, parent-reported home environment, and openness. In this cohort, too, the change in cognitive abilities was largely associated with previous cognitive performance at the age of 17. In addition, PGS_CA_ was partly related to the SES differences in cognitive abilities.

When interpreting these results, it should be noted that some of the variables examined are closely intertwined with SES through common familial and genetic background processes (parents’ cognitive abilities and PGS_CA_), so that the underlying causal mechanisms linking SES to cognition cannot be clearly disentangled. Their inclusion helps to contextualise the SES–cognition relationship, rather than to identify indirect causal pathways. The longitudinal design furthermore made it possible to show that these factors are associated with children’s cognitive abilities not only at the first measurement point, but over a period of six years.

Almost all variables correlated as expected with the cognitive ability of children and parental SES, e.g., PGS_CA_ (Demange et al., 2021; Gorelik et al., 2024; Lee et al., 2018). However, the magnitude of the correlation with the predictors of home environment of children and parents and openness was—at least for some cohorts and time points—lower than in previous research (Anglim et al., 2022; Deater-Deckard et al., 2009; Hart et al., 2007; Lechner et al., 2017). This may be due to previous studies being based on US samples (e.g., Petrill et al., 2004; Hart et al., 2007), whereas the present study derived data from a German cohort, and the effects may differ between these two countries. However, another study also found no correlation between children’s cognitive ability and the home environment in a sample with the same age range as cohort 1 (Dumas et al., 2005). In contrast to previous research, reading to the child was not associated with a child’s cognitive ability in the analyses despite some small correlation (McGinnity et al., 2022; Schneider & Linberg, 2022; Sullivan & Brown, 2013). This could be due to the fact that in previous research mostly language-related skills were assessed. In addition, Linberg et al. (2019) showed that while there was an effect for language skills, there was no effect for mathematical skills, which could suggest that reading to the child may be less important for a child’s general cognitive skills. It is also possible that the factor could not make an independent contribution beyond the other variables. As in previous studies, maternal cognitive ability affected a child’s cognitive ability even after taking into account parental SES (Eriksen et al., 2013; Ronfani et al., 2015; Tong et al., 2007; Torres, 2013), and the association between SES and cognitive abilities decreased considerably after including parental cognitive ability (Marks & O’Connell, 2023; Tong et al., 2007; Torres, 2013). In addition, the finding that maternal cognitive abilities are associated with SES differences in more scholastic cognitive abilities (Marks & O’Connell, 2023) could also be replicated using a fluid cognition measure and parallel assessments for parents and children. Besides maternal cognitive ability, we also found effects for paternal cognitive ability (Anger & Heineck, 2010).

The key research findings on which this study was based are the increasing SES disparities in children’s cognitive abilities with age, which were found in two longitudinal studies (Feinstein, 2003; von Stumm & Plomin, 2015). This finding was replicated in this study with a German sample, suggesting that the increasing SES disparities may not be limited to British cohorts. In addition, previous studies have measured preschool children’s cognitive abilities in various ways, some of which could be categorised more as developmental measurements (e.g., language development and fine motor skills), with the longitudinal measures varying to some degree between measurement time points (e.g., Feinstein, 2003; von Stumm & Plomin, 2015). As a result, the longitudinal measurements may be more difficult to compare. To avoid these difficulties, a harmonised measurement of fluid cognitive ability was used in the present study. Overall, the magnitude of the longitudinal correlations of cognitive ability and SES at different age stages was comparable to the results of von Stumm and Plomin (2015), which could strengthen the generalisability of the findings. In addition, the stability of cognitive ability increased with age (Breit et al., 2024), suggesting that possible interventions could be most effective at an earlier age.

In general, the results suggest that the SES gap in cognitive ability primarily emerges between the ages of 5 and 11. Comparing the results across cohorts and time points of the analyses without parental cognitive ability in order to better assess the overall SES effect (Supplementary Tables S4 and S5), SES-related differences appeared to increase more strongly in cohort 1 from F2F 1 to F2F 4 (from β = 0.10 to β = 0.27), but from then on remained more stable until the age of 23 (β = 0.27), although at age 23 the results may still suggest slight SES effects. In the study by von Stumm and Plomin (2015), the greatest leap in the SES disparities was observable between the ages of 4 and 7, which in turn indicates that the SES differences rise during primary school. The start of schooling could place a stronger focus on the child’s education than was the case in kindergarten or preschool, which means that parents with higher SES could begin to support their children even more in primary school, for example through cognitive stimulation (Lurie et al., 2021; Rakesh et al., 2024). In addition, cognitive ability at age 5 was more weakly associated with cognitive ability at age 11 compared to the other, older cohorts, which may reflect that children’s cognitive development processes are more variable in the earlier school years and could be especially receptive to parental or environmental influence. Even though results and correlations with regard to parental SES appeared to be descriptively comparable across the three age cohorts, the graphs in Figure 2 do not fully intersect, meaning that cohort effects cannot be ruled out.

As important as these results are, after including parental cognitive ability, the association between SES and cognitive abilities decreased considerably. In most analyses, the SES effect was rather small and strongly linked to academic track attendance or prior cognitive ability. Parental cognitive ability was robustly associated with cognitive ability and the change in cognitive ability in the two younger cohorts. Since the cognitive ability of parents determines their education and thus also their occupation and income, it is quite reasonable that parental SES is also strongly linked to the cognitive ability of the children. The indirect effect indicates that the SES-related differences in the children’s cognitive ability may reflect different underlying conditions. On the one hand, it is more likely that parents with higher cognitive ability pass on the corresponding genetic predispositions; on the other hand, these parents may also be better able to stimulate their children cognitively through interactions and the environment in which they grow up. The higher cognitive ability of the parents, which was strongly associated with the SES differences, can presumably not be explained exclusively by genetic factors, as SES effects have also been found in adoption studies (Kendler et al., 2015). This suggests that parents with higher cognitive ability might also exhibit behaviours that positively influences their children’s cognitive abilities. The exact behaviours could not be identified in this study and should be examined more closely in future research. For example, it could be assumed that parents with higher cognitive abilities may be more successful in terms of cognitive stimulation (Lurie et al., 2021; Rakesh et al., 2024) or learning environment (Junge et al., 2021), so that their children could benefit more from schooling. However, since parental cognitive abilities and parental SES are closely intertwined, the exact processes through which parents’ cognitive abilities are linked to their children’s cognitive abilities cannot be precisely determined in this study and require further investigation. Since the cognitive ability of parents was associated with cognitive abilities beyond parental SES, children from all SES backgrounds tended to have higher cognitive scores if their parents displayed higher cognitive abilities.

In line with the increasing SES differences in cognitive abilities, associations with paternal cognitive ability were strongest between the ages of 5 and 11. In summary, maternal and paternal cognitive ability were related to cognitive ability from the age of 5 and up to the age of 17. Finally, from the age of 23, these associations were no longer observed. This finding could reflect either that children no longer lived in the household and could therefore no longer be actively supported, or simply that the stability of cognitive ability increases with age (Breit et al., 2024) and the parental component may already have fully exerted its effect by this age. In addition, academic track attendance in secondary school appeared to be related to SES, as SES was no longer significant in a number of analyses after controlling for this factor. In exploratory analyses (Supplementary Tables S6 and S7), academic track attendance was linked to the SES-associated differences in cognitive ability. This finding suggests that children from high SES families are more likely to attend the academic track than children from low SES families, which might be associated with higher gains in cognitive abilities and thus with SES-related differences in cognitive abilities. The allocation of children to the academic track, which in Germany is based on the educational recommendations given by teachers, differs by SES: children with a lower SES receive poorer educational recommendations than children with a higher SES, despite displaying the same school performance or cognitive ability level (Arnold et al., 2007; Paulus et al., 2021). The fact that children attending the academic track tend to show larger increases in cognitive scores (Becker et al., 2012) could contribute to disparities between SES groups. However, since academic track attendance is influenced by both prior cognitive ability and SES, the underlying mechanisms cannot be clearly established. The temporal ordering of measurements in the youngest cohort allows for a more cautious associative interpretation, but selection effects due to SES cannot be completely ruled out. As the analysis was exploratory, the results should be interpreted with caution but may provide useful insights for future research.

While the effect of PGS_CA_ on cognitive performance was somewhat inconsistent (though significant in some models) up to the age of 17, a more stable association emerged at age 23. This pattern is consistent with previous findings from twin studies, where genetic influence on cognitive ability has been shown to increase with age (Haworth et al., 2010) and which have also been found in a nuclear twin family design using the TwinLife sample (Wolfram et al., 2024). One possible explanation is that individuals may increasingly select or shape their environments in ways that align with traits associated with the polygenic score, such that cumulative gene–environment correlation (r_GE_) contributes to the observed effect. It is important to note, however, that PGS are based on genome-wide association studies typically conducted in adult populations, and their predictive power may reflect age-specific influences, including differences in the trait as measured in children versus adults or the developmental timing of relevant genetic pathways. By age 23, the PGS_CA_ was associated not only with prior cognitive performance at age 17 but also with change over time, indicating an indirect effect of SES at this age. Notably, PGS_CA_ was also associated with children’s cognitive abilities when parental cognitive abilities were included as a predictor. While this may reflect effects not fully captured by parental phenotype, such findings must be interpreted with caution, as population-level PGS associations can be influenced by stratification, indirect genetic effects, or differences in environmental exposures. However, in the exploratory analyses, in which the PGS_CA_ of the parents was controlled for (Supplementary Tables S8 and S9), the PGS_CA_ of the children and adolescents in this cohort still showed a significant association of the same magnitude as in the longitudinal regression analyses. These analyses, using parental PGS as a control variable, implicate that the effect of PGS_CA_ may in fact be a direct genetic effect and is not caused by, e.g., indirect genetic effects. Overall, the inclusion of the PGS_CA_ adds valuable explanatory power and contributes to understanding how genomic associations with cognitive performance may manifest across development and how they relate to SES. However, in order to fully account for assortative mating, population stratification, and genetic nurture, a within-family PGS specification should be performed in a subsequent study (Selzam et al., 2019). 

For the children of the youngest cohort, openness to experience was partly linked to SES-related differences in cognitive abilities, even when all control factors were considered. Since one facet of the personality factor openness is intellectual curiosity (Anglim et al., 2022), it is plausible that children with higher levels of openness engage in experiences related to their intellectual curiosity, which may be associated with better cognitive development (Staff et al., 2017). In this context, a suitable approach could be to provide extracurricular learning opportunities for all children as early as primary school, which might also promote openness among children with lower SES and fewer resources. However, for cohort 2, openness showed no association, so that these results should be interpreted with caution. For cohort 3, the relation to cognitive abilities was only present when previous cognitive performance was not controlled for. The precise effect should be investigated in more detail in a future study.

### 4.1. Limitations

The main limitation of our study is the inclusion of three different age cohorts. This means that we cannot depict the precise course of the SES gap in cognitive ability over the age range from 5 to 23 years. Cohort effects may occur, leading to differences that are not caused by the factors analysed here and therefore limiting comparability across the entire age range. However, an age overlap enables the correlations to be compared for two age points (11 and 17 years) between the cohorts and indicates that the associations at these age points were similar in magnitude. Nevertheless, we would recommend analysing the SES-related differences over a larger age range with a single cohort and multiple measurement points to more precisely identify developmental trajectories. In addition, merely two measurement points for cognitive ability were available for each cohort, which required the use of a residual change model. Although latent factor scores were used to reduce measurement error at each time point, residual change models do not explicitly model errors in the change itself. Therefore, the estimated effects may still be less precise than estimates from a latent growth curve model (Castro-Schilo & Grimm, 2018). It is also possible that the observed cognitive change may partly reflect regression to the mean rather than true cognitive changes (Barnett et al., 2005). Thus, replicating the analyses using latent change score models with more than two measurement points would be beneficial in future studies.

As parental SES does not vary within families, within-family analyses of SES effects were not feasible. The between-family approach, even though it is applied longitudinally in a residual change model, cannot fully account for prior influencing factors, including unobserved environmental or genetic effects. Moreover, the study design does not allow causal pathways to be disentangled from selection processes. Accordingly, the results should be interpreted only as indicative of potential pathways and not as definitive causal relationships.

In addition, for the youngest cohort, the CFT 20-R at age 5 and the CFT 1-R at age 11 were not age-appropriate, so this cohort completed two different versions of the CFT. Although both tests are grounded in the same framework and measure fluid cognitive abilities (Weiß, 2006; Weiß & Osterland, 2013), the two tests differed in the number of subtests, the total items, and the item difficulty. To account for this, separate factor analyses were conducted for each time point so that the results of both tests could be put in relation to each other. Furthermore, three of the subtests of each cognition test were structured in parallel and showed metric invariance (ΔCFI < 0.01; Chen, 2007), indicating that both test forms share an equivalent factor structure with comparable factor loadings. However, since the two test versions differ in terms of item difficulty, analyses of longitudinal comparison should be interpreted with caution. Due to the non-equivalence of the tests, the comparability of cognitive change within the youngest cohort, as well as comparisons with the older cohorts, could be limited and may partly reflect measurement differences rather than developmental differences. Besides the CFT, the validity of the PGS could also be limited. The variants included in the calculation of the PGS are genetic correlates of the trait as well as spuriously associated variants. Although the PGS is predictive, the extent to which the variants exert a direct genetic effect on the trait has yet to be determined.

Since TwinLife is a comprehensive longitudinal study, this comes with some disadvantages. Firstly, parents with a higher education are somewhat overrepresented, so that parental SES could be affected by range restriction, potentially leading to an underestimation of SES effects (Lang & Kottwitz, 2017). Secondly, non-random dropout rates may occur in longitudinal studies. Even though we analysed the sample in this respect and included auxiliary variables, this non-random dropout could lead to biased estimates. As sensitivity analyses, analyses with only available data on the second measurement point of cognitive ability and analyses using inverse probability weighting of selective panel dropout (Krell & Ruks, 2023) were conducted and included in the Supplementary Material for comparison, whereby the results of the study appeared robust (Supplementary Tables S10–S13). Thirdly, some constructs were measured using short scales with lower internal consistency (e.g., home environment α = 0.54–0.65) as well as “reading to the child” using a single item, which could increase the risk of random measurement errors and limit construct validity, so that the effects could be underestimated in the present study. Finally, TwinLife only contains twin families, which means that results could not necessarily be generalised to the general population in every respect. For example, in relation to our research question, the parental capacity to promote cognitive abilities may be more limited as parents are caring for two children of the same age. Overall, while the overrepresentation of high SES families and the use of short scales could lead to an underestimation of effect sizes, the non-random dropout and the inclusion of twin families could result in the underestimation or overestimation of effect sizes. These limitations should be considered when interpreting the results, as they may limit the generalisability of the findings.

### 4.2. Conclusions

In summary, this study showed that parental SES is linked to differences in cognitive abilities and that these SES-related differences emerge mainly between the ages of 5 and 11. However, SES remained associated with cognitive abilities beyond this age, up to the age of 23, reflecting a further widening of the observed gap. By including the cognitive abilities of parents, this study indicates that—in contrast to educational inequality—the SES differences in children’s cognitive abilities appear more equitable. Nevertheless, the findings suggest that attending the academic track may be associated with inequalities, as previous research has shown that these are allocated unequally according to SES.

## Figures and Tables

**Table 1:** Descriptive statistics for cognitive ability, parental SES, home environment, reading to the child (only C1), PGS_CA_, openness, and the control variables age, academic track, and years of education.

	Manifest Factor									Latent Factor					
	Cohort 1			Cohort 2			Cohort 3			Across All Cohorts		Cronbach’s α			
	*M*	*SD*	*N*	*M*	*SD*	*N*	*M*	*SD*	*N*	*M*	*SD*	C1	C2	C3	P
Age F2F 1	5.49	0.38	2100	11.45	0.40	2273	17.48	0.41	2342	-	-	-	-	-	-
Age F2F 4	11.51	0.36	1290	17.45	0.40	1272	23.49	0.38	1028	-	-	-	-	-	-
CA F2F 1 *	17.58	6.86	1678	32.33	7.22	2153	39.99	7.55	2252	*0.00*	*0.86*	0.70	0.69	0.79	-
CA F2F 4	34.69	7.49	705	41.94	7.65	764	44.52	6.68	695	*0.00*	*0.90*	0.72	0.80	0.77	-
SES	-	-	-	-	-	-	-	-	-	0.06	0.95	-	-	-	0.56
CA Mother	37.66	7.95	961	37.18	8.02	1092	36.00	7.83	1070	0.00	0.90	-	-	-	0.79
CA Father	39.73	7.93	704	38.99	8.37	737	37.69	8.47	689	0.00	0.90	-	-	-	0.80
HE Child	17.85	3.98	1376	19.45	3.65	2031	19.04	3.66	2262	*−0.01*	*0.81*	0.54	0.62	0.65	-
HE Mother	19.81	3.16	992	20.53	3.20	1114	20.42	3.19	1121	−0.03	0.82	-	-	-	0.61
HE Father	19.83	3.03	733	20.28	2.99	792	20.32	3.01	745	−0.02	0.82	-	-	-	0.56
Reading	4.32	0.84	2067	-	-	-	-	-	-	-	-	-	-	-	-
PGS_CA_	-	-	877	-	-	923	-	-	735	0.00	0.24	-	-	-	-
Openness	14.27	3.97	992	15.61	3.64	2230	14.45	3.53	2316	*−0.01*	*0.72*	0.63	0.51	0.58	-
Years of education	-	-	-	-	-	-	13.85	2.27	966						
	Yes		No	Yes		No	Yes		No						
Academic track	51.4%		48.6%	46.6%		53.4%	54.2%		45.8%						

Note. A total of 6715 individuals (53.3% female) and 6291 parents (=P) in 3178 families were included in the analysis. Descriptive statistics for the manifest factors were calculated using mean values (=*M*) and standard deviations (=*SD*) of the sum score. Scores for CA ranged from 8 to 55 (for CFT 20-R; for CFT 1-R: 2–43), for reading to the child from 1 to 5, for openness (for C1 in F2F 4) from 3 to 21, and for HE from 5 to 25. Since the latent factors were very similar across cohorts, we only report the descriptive statistics for C2 (in italics). CA = cognitive ability, HE = home environment, C1 = cohort 1, C2 = cohort 2, and C3 = cohort 3. * for C1: CFT 1-R.

**Table 2:** Correlations for cognitive ability, parental SES, home environment (children and parents), reading to the child (only C1), PGS_CA_, and openness, separately for each cohort.

	**Cohort 1**	**2**	**3**	**4**	**5**	**6**	**7**	**8**	**9**	**10**
1	CA F2F 1	0.22 ***	0.13 ***	0.20 ***	0.18 ***	0.00	0.05	0.06 *	0.09 *	0.05
2	CA F2F 4		0.35 ***	0.32 ***	0.40 ***	−0.02	0.02	0.15 **	0.17 **	0.17 ***
3	SES			0.52 ***	0.52 ***	0.12 **	0.07 *	0.41 ***	0.21 ***	0.19 ***
4	CA Mother				0.44 ***	0.05	−0.02	0.29 ***	0.18 **	0.06
5	CA Father					0.07	−0.06	0.26 ***	0.26 ***	0.15 **
6	HE Child						0.25 ***	0.20 ***	0.05	0.11 **
7	HE Parents							0.11 **	0.03	0.15 ***
8	Reading								0.11 *	0.12 **
9	PGS_CA_									0.15 **
10	Openness									
	**Cohort 2**	**2**	**3**	**4**	**5**	**6**	**7**	**8**	**9**	**10**
1	CA F2F 1	0.63 ***	0.33 ***	0.38 ***	0.38 ***	0.10 ***	0.08 **	-	0.21 ***	0.06 *
2	CA F2F 4		0.33 ***	0.40 ***	0.36 ***	0.14 ***	0.07	-	0.18 ***	0.08 *
3	SES			0.49 ***	0.54 ***	0.13 ***	0.04	-	0.18 **	0.05 *
4	CA Mother				0.35 ***	0.08 **	0.00	-	0.20 ***	0.07 **
5	CA Father					0.10 **	0.01	-	0.20 ***	0.00
6	HE Child						0.33 ***	-	0.00	0.09 ***
7	HE Parents							-	−0.04	0.04
8	Reading								-	-
9	PGS_CA_									0.05
10	Openness									
	**Cohort 3**	**2**	**3**	**4**	**5**	**6**	**7**	**8**	**9**	**10**
1	CA F2F 1	0.72 ***	0.34 ***	0.39 ***	0.40 ***	0.07 **	0.13 ***	-	0.25 ***	0.09 ***
2	CA F2F 4		0.37 ***	0.37 ***	0.37 ***	0.08 *	0.16 ***	-	0.31 ***	0.09 *
3	SES			0.45 ***	0.49 ***	0.19 ***	0.20 ***	-	0.23 ***	0.08 ***
4	CA Mother				0.35 ***	0.07 **	0.10 **	-	0.27 ***	0.06 *
5	CA Father					0.09 **	0.07	-	0.14	0.04
6	HE Child						0.38 ***	-	0.04	0.03
7	HE Parents							-	0.08	0.01
8	Reading								-	-
9	PGS_CA_									−0.00
10	Openness									

Note. * *p* < .05, ** *p* < .01, *** *p* < .001. CA = cognitive ability, HE = home environment, F2F 1 = first face-to-face interview, F2F 4 = fourth face-to-face interview, C1 = cohort 1, C2 = cohort 2, and C3 = cohort 3.

**Table 3:** Standardised regression estimates (β) of the multiple regression analyses for the outcome cognitive ability at F2F 1, separately for each cohort.

	Cohort 1	Cohort 2		Cohort 3	
		Model 1	Model 2	Model 1	Model 2
SES	−0.01	0.07 *	−0.01	0.06 *	0.02
CA Mother	0.15 ***	0.25 ***	0.20 ***	0.22 ***	0.20 ***
CA Father	0.10 *	0.22 ***	0.21 ***	0.26 ***	0.24 ***
HE—Child	−0.02	0.02	0.01	−0.02	−0.03
HE—Parents	0.07 *	0.07 **	0.07 **	0.07 **	0.06 *
Reading to the child	−0.00	-	-	-	-
PGS_CA_	0.05	0.11 **	0.10 **	0.14 **	0.10 *
Openness	-	0.03	0.02	0.06 **	0.06 **
Age	0.18 ***	0.07 **	0.08 ***	0.08 ***	0.07 ***
Sex	0.05	0.04 *	0.03	−0.05 *	−0.06 **
Birth weight	0.10 ***	0.09 ***	0.07 ***	−0.02	−0.02
Academic track	-	-	0.26 ***	-	0.22 ***
SOEP years of education	-	-	-	-	-
*R* ^2^	9.7% ***	24.9% ***	30.0% ***	27.0% ***	31.0% ***

Note. * *p* < .05, ** *p* < .01, *** *p* < .001. CA = cognitive ability, HE = home environment, F2F 1 = first face-to-face interview, F2F 4 = fourth face-to-face interview, C1 = cohort 1, C2 = cohort 2, and C3 = cohort 3.

**Table 4:** Standardised regression estimates (β) of the multiple regression analyses for the outcome cognitive ability at F2F 4 (second cognitive ability measurement), separately for each cohort.

	Cohort 1			Cohort 2			Cohort 3		
	Model 1	Model 2	Model 3	Model 1	Model 2	Model 3	Model 1	Model 2	Model 3
SES	0.11 *	0.12 *	0.07	0.06	0.02	−0.03	0.10 *	0.06	0.05
CA Mother	0.14 **	0.12 *	0.11 *	0.28 ***	0.15 ***	0.13 ***	0.17 ***	0.04	0.03
CA Father	0.25 ***	0.23 ***	0.22 ***	0.22 ***	0.10	0.10 *	0.22 ***	0.05	0.03
HE—Child	−0.07 *	−0.07	−0.06	0.07 *	0.06	0.05	−0.03	−0.02	−0.02
HE—Parents	0.04	0.03	0.02	0.04	0.01	0.01	0.09 *	0.04	0.04
Reading to the child	−0.01	−0.02	−0.02	-	-	-	-	-	-
PGS_CA_	0.06	0.05	0.04	0.07	0.01	0.02	0.21 ***	0.12 **	0.10 **
Openness	0.10 **	0.10 **	0.10 **	0.05	0.03	0.03	0.07 *	0.03	0.03
Age	0.03	0.01	0.01	−0.00	−0.03	−0.03	0.02	−0.02	−0.02
Sex	−0.04	−0.05	−0.05	−0.01	−0.04	−0.04	−0.16 ***	−0.13 ***	−0.13 ***
Birth weight	0.08 *	0.06	0.05	−0.02	−0.06 *	−0.07 *	−0.02	−0.01	−0.01
CA F2F 1	-	0.13 **	0.11 **	-	0.53 ***	0.49 ***	-	0.61 ***	0.60 ***
Academic track	-	-	0.14 **	-	-	0.16 ***	-	-	0.09 *
SOEP years of education	-	-	-	-	-	-	-	-	0.03
*R* ^2^	21.1% ***	22.5% ***	23.2% ***	23.9% ***	45.3% ***	46.8% ***	28.6% ***	56.1% ***	56.9% ***

Note. * *p* < .05, ** *p* < .01, *** *p* < .001. CA = cognitive ability, HE = home environment, F2F 1 = first face-to-face interview, F2F 4 = fourth face-to-face interview, C1 = cohort 1, C2 = cohort 2, and C3 = cohort 3.

**Table 5:** Standardised regression estimates (β) (and unstandardised total effects) of the decomposition analyses for the outcome cognitive ability at F2F 1, separately for each cohort.

	Cohort 1		Cohort 2				Cohort 3			
			Model 1		Model 2		Model 1		Model 2	
	Direct Effect	Indirect Effect	Direct Effect	Indirect Effect	Direct Effect	Indirect Effect	Direct Effect	Indirect Effect	Direct Effect	Indirect Effect
CA Mother	0.15 ***	0.08 ***	0.25 ***	0.12 ***	0.20 ***	0.10 ***	0.22 ***	0.10 ***	0.20 ***	0.09 ***
CA Father	0.10 *	0.05 *	0.22 ***	0.12 ***	0.21 ***	0.11 ***	0.26 ***	0.13 ***	0.24 ***	0.12 ***
HE—Child	−0.02	−0.00	0.02	0.00	0.01	0.00	−0.02	−0.00	−0.03	−0.00
HE—Parents	0.07 *	0.00	0.07 **	0.00	0.07 **	0.00	0.07 **	0.01 *	0.06 *	0.01 *
Reading to the child	−0.00	0.00	-	-	-	-	-	-	-	-
PGS_CA_	0.05	0.01	0.11 **	0.02 *	0.10 **	0.02 *	0.14 **	0.03 *	0.10 *	0.02
Openness	-	-	0.03	0.00	0.02	0.00	0.06 **	0.01 *	0.06 **	0.00 *
Age	0.18 ***		0.07 **		0.08 ***		0.08 ***		0.07 **	
Sex	0.05		0.04 *		0.03		−0.05 *		−0.06 **	
Birth weight	0.10 ***		0.09 ***		0.07 ***		−0.02		−0.02	
Academic track	-		-		0.26 ***		-		0.22 ***	
Direct effect of SES	−0.01		0.07 *		−0.01		0.06 *		0.02	
Total indirect effect	0.12 ***		0.24 ***		0.22 ***		0.27 ***		0.24 ***	
Total effect of SES	0.11 ***		0.30 ***		0.20 ***		0.33 ***		0.25 ***	
*R* ^2^	9.7% ***		24.9% ***		30.0% ***		27.0% ***		31.0% ***	

Note. * *p* < .05, ** *p* < .01, *** *p* < .001. CA = cognitive ability, HE = home environment, F2F 1 = first face-to-face interview, F2F 4 = fourth face-to-face interview, C1 = cohort 1, C2 = cohort 2, and C3 = cohort 3.

**Table 6:** Standardised regression estimates (β) (and unstandardised total effects) of the decomposition analyses for the outcome change in cognitive ability.

	Cohort 1		Cohort 2		Cohort 3	
	Direct Effect	Indirect Effect	Direct Effect	Indirect Effect	Direct Effect	Indirect Effect
CA F2F 1	0.11 **	0.01 *	0.49 ***	0.16 ***	0.60 ***	0.20 ***
CA Mother	0.11 *	0.06 *	0.13 ***	0.07 ***	0.03	0.01
CA Father	0.22 ***	0.11 ***	0.10 *	0.05 *	0.03	0.02
HE—Child	−0.06	−0.00	0.05	0.01	−0.02	−0.00
HE—Parents	0.02	0.00	0.01	0.00	0.04	0.01
Reading to the child	−0.02	−0.00	-	-	-	-
PGS_CA_	0.04	0.01	0.02	0.00	0.10 *	0.02 *
Openness	0.10 **	0.02 *	0.03	0.00	0.03	0.00
Age	0.01		−0.03		−0.02	
Sex	−0.05		−0.04		−0.13 ***	
Birth weight	0.05		−0.07 *		−0.00	
Academic track	0.14 **		0.16 ***		0.09 *	
SOEP years of education	-		-		0.03	
Direct effect of SES	0.07		−0.03		0.05	
Total indirect effect	0.17 ***		0.30 ***		0.29 ***	
Total effect of SES	0.24 ***		0.26 ***		0.34 ***	
*R* ^2^	23.2% ***		46.8% ***		56.9% ***	

Note. * *p* < .05, ** *p* < .01, *** *p* < .001. CA = cognitive ability, HE = home environment, F2F 1 = first face-to-face interview, F2F 4 = fourth face-to-face interview, C1 = cohort 1, C2 = cohort 2, and C3 = cohort 3.

## Data Availability

All data and research materials can be accessed via Gesis. The TwinLife data (Diewald et al., 2024) is available for research purposes free of charge after signing a data use agreement. Please see https://dbk.gesis.org/dbksearch/SDesc2.asp?DB=D&no=6701 (accessed on 20 February 2025) for more information on the TwinLife data. At the time of this study, however, the genetic data is not available via Gesis. The analysis code is available in the Supplementary Material.

## Notes

1 Please note that a PGS reflects a statistical association with a phenotype at the population level and cannot be used to make claims about individuals.

2 According to the preregistration, we initially planned to calculate single mediation models first, followed by an overall model that includes the significant mediators. However, due to statistical considerations (suppressor effects, inflation of type 1 errors), we opted for an overall model directly instead.

3 To adequately take the clustering of the twins and siblings into account, the Mplus option cluster = familyID and type = complex was used.

4 See https://dbk.gesis.org/dbksearch/SDesc2.asp?DB=D&no=6701 (accessed on 20 February 2025) for more information on the TwinLife data. At the time of this study, however, the genetic data is not available via Gesis.

## Supplementary Materials

The following supporting information can be downloaded at: https://www.mdpi.com/article/10.3390/jintelligence14040063/s1, Table S1: Standardized regression estimates (β) (and unstandardized total effects) of the decomposition analyses for the outcome cognitive ability at F2F 4 or change in cognitive ability, Cohort 1; Table S2: Standardized regression estimates (β) (and unstandardized total effects) of the decomposition analyses for the outcome cognitive ability at F2F 4 or change in cognitive ability, Cohort 2; Table S3: Standardized regression estimates (β) (and unstandardized total effects) of the decomposition analyses for the outcome cognitive ability at F2F 4 or change in cognitive ability, Cohort 3; Table S4: Standardized regression estimates (β) of the multiple regression analyses for the outcome cognitive ability at F2F 1, separately for each cohort, excluding the predictors maternal and paternal cognitive ability; Table S5: Standardized regression estimates (β) of the multiple regression analyses for the outcome change in cognitive ability, separately for each cohort, excluding the predictors maternal and paternal cognitive ability; Table S6: Explanatory standardized regression estimates (β) (and unstandardized total effects) of the decomposition analyses for the outcome cognitive ability at F2F 1 including academic track attendance, separately for each cohort (CA = cognitive ability, HE = home environment, F2F1 = first face-to-face interview); Table S7: Explanatory standardized regression estimates (β) (and unstandardized total effects) of the decomposition analyses for the outcome change in cognitive ability including academic track attendance and years of education, separately for each cohort (CA = cognitive ability, HE = home environment, F2F1 = first face-to-face interview); Table S8: Standardized regression estimates (β) of the multiple regression analyses for the outcome cognitive ability at F2F 1, separately for each cohort, including the predictors maternal and paternal PGSCA; Table S9: Standardized regression estimates (β) of the multiple regression analyses for the outcome cognitive ability at F2F 4, separately for each cohort, including the predictors maternal and paternal PGSCA; Table S10: Standardized regression estimates (β) of the multiple regression analyses for the outcome change in cognitive ability, only cases with available data on CA F2F 4; Table S11: Standardized regression estimates (β) (and unstandardized total effects) of the decomposition analyses for the outcome change in cognitive ability, only cases with available data on CA F2F 4; Table S12: Standardized regression estimates (β) of the multiple regression analyses for the outcome cognitive ability at F2F 4 (second cognitive ability measurement) using inverse probability weighting of selective panel dropout (CA = cognitive ability, HE = home environment, F2F1 = first face-to-face interview, F2F4 = fourth face-to-face interview); Table S13: Standardized regression estimates (β) (and unstandardized total effects) of the decomposition analyses for the outcome change in cognitive ability using inverse probability weighting of selective panel dropout (CA = cognitive ability, HE = home environment, F2F1 = first face-to-face interview, F2F4 = fourth face-to-face interview). Syntax 1. Multiple regression analysis, exemplary for cohort 1 and model 3, outcome change in cognitive ability; Syntax 2. Decomposition analysis, exemplary for cohort 3, outcome change in cognitive ability.
