# Supplementary material for: Socioeconomic Differences in Cognitive Ability Across Childhood and Adolescence: An Investigation of Genetic, Individual, and Environmental Factors
Source: J Intell. 2026 Apr 10;14(4):63. doi: 10.3390/jintelligence14040063 (PMC13118255; doi:10.3390/jintelligence14040063)
Supplement: Supplementary file 1 [file jintelligence-14-00063-s001.zip › Supplementary Material_24.03.2026.pdf]

Supplementary material

Tables S1-S3	All models of the decomposition analyses for the outcome change in cognitive ability, separately for each cohort.
Table S4	Multiple regression analyses for the outcome cognitive ability at F2F 1, excluding parental cognitive ability.
Table S5	Multiple regression analyses for the outcome change in cognitive ability, excluding parental cognitive ability.
Table S6	Decomposition analysis for the outcome cognitive ability at F2F 1, including track attendance
Table S7	Decomposition analysis for the outcome change in cognitive ability, including track attendance (and, for cohort 3, years of education)
Table S8	Multiple regression analyses for the outcome cognitive ability at F2F 1, including the predictors maternal and paternal PGS_{CA}.
Table S9	Multiple regression analyses for the outcome change in cognitive ability, including the predictors maternal and paternal PGS_{CA}.
Table S10	Robustness check of the multiple regression analyses for the outcome change in cognitive ability; only cases with available data on cognitive ability at F2F 4
Table S11	Robustness check of the decomposition analyses for the outcome change in cognitive ability; only cases with available data on cognitive ability at F2F 4
Table S12	Robustness check of the multiple regression analyses for the outcome change in cognitive ability using inverse probability weighting of selective panel dropout
Table S13	Robustness check of the decomposition analyses for the outcome change in cognitive ability using inverse probability weighting of selective panel dropout

Table S1

Standardized regression estimates (β) (and unstandardized total effects) of the decomposition analyses for the outcome cognitive ability at F2F 4 or change in cognitive ability, Cohort 1.

	Cohort 1					
	Model 1		Model 2		Model 3	
	Direct effect	Indirect effect	Direct effect	Indirect effect	Direct effect	Indirect effect
CA F2F 1	-	-	0.13**	0.02**	0.11**	0.01*
CA Mother	0.14**	0.07**	0.12*	0.06*	0.11*	0.06*
CA Father	0.25***	0.13***	0.23***	0.12***	0.22***	0.11***
HE - Child	-0.07*	-0.01	-0.07	-0.01	-0.06	-0.00
HE - Parents	0.04	0.00	0.03	0.00	0.02	0.00
Reading to the child	-0.01	-0.01	-0.02	-0.01	-0.02	-0.00
PGS_{CA}	0.06	0.01	0.05	0.01	0.04	0.01
Openness	0.10**	0.02*	0.10**	0.02*	0.10**	0.02*
Age	0.03		0.01		0.01	
Sex	-0.04		-0.05		-0.05	
Birth weight	0.08*		0.06		0.05	
Academic track	-	-	-	-	0.14**	
					-	-
Direct effect of SES	0.11*		0.12*		0.07	
Total indirect effect	0.20***		0.19***		0.17***	
Total effect of SES	0.30***		0.30***		0.24***	
R^2	21.1%***		22.5%***		23.2%***	

Note. *$p < .05$, **$p < .01$, ***$p < .001$

Table S2

Standardized regression estimates (β) (and unstandardized total effects) of the decomposition analyses for the outcome cognitive ability at F2F 4 or change in cognitive ability, Cohort 2.

	Cohort 2					
	Model 1		Model 2		Model 3	
	Direct effect	Indirect effect	Direct effect	Indirect effect	Direct effect	Indirect effect
CA F2F 1	-	-	0.53***	0.18***	0.49***	0.16***
CA Mother	0.24***	0.12***	0.15***	0.07***	0.13***	0.07***
CA Father	0.24***	0.13***	0.10	0.05	0.10*	0.05*
HE - Child	0.07	0.01	0.06	0.01	0.05	0.01
HE - Parents	0.07	0.00	0.01	0.00	0.01	0.00
PGS_{CA}	0.08	0.01	0.01	0.00	0.02	0.00
Openness	0.05	0.00	0.03	0.00	0.03	0.00
Age	0.01		-0.03		-0.03	
Sex	-0.03		-0.04		-0.04	
Birth weight	-0.04		-0.06*		-0.07*	
Academic track	-	-	-	-	0.16***	
Direct effect of SES	0.04		0.02		-0.03	
Total indirect effect	0.28***		0.32***		0.30***	
Total effect of SES	0.31***		0.34***		0.26***	
R^2	22.5%***		45.3%***		46.8%***	

Note. $^*p < .05$, $^{**}p < .01$, $^{***}p < .001$

Table S3

Standardized regression estimates (β) (and unstandardized total effects) of the decomposition analyses for the outcome cognitive ability at F2F 4 or change in cognitive ability, Cohort 3.

	Cohort 3					
	Model 1		Model 2		Model 3	
	Direct effect	Indirect effect	Direct effect	Indirect effect	Direct effect	Indirect effect
CA F2F 1	-	-	0.61***	0.21***	0.60***	0.20***
CA Mother	0.14**	0.06**	0.04	0.02	0.03	0.01
CA Father	0.16**	0.08**	0.05	0.03	0.03	0.02
HE - Child	-0.02	-0.00	-0.02	-0.00	-0.02	-0.00
HE - Parents	0.06	0.01	0.04	0.01	0.04	0.01
PGS_{CA}	0.18**	0.04**	0.12**	0.03**	0.10*	0.02*
Openness	0.08*	0.01	0.03	0.00	0.03	0.00
Age	-0.02		-0.02		-0.02	
Sex	-0.19***		-0.13***		-0.13***	
Birth weight	-0.04		-0.01		-0.00	
Academic track	-	-	-	-	0.09*	
SOEP years of education	-	-	-	-	0.03	
Direct effect of SES	0.15**		0.06		0.05	
Total indirect effect	0.19***		0.31***		0.29***	
Total effect of SES	0.34***		0.38***		0.34***	
R^2	25.3%***		56.1%***		56.9%***	

Note. **p < .05, **p < .01, ***p < .001*

Table S4

Standardized regression estimates (β) of the multiple regression analyses for the outcome cognitive ability at F2F 1, separately for each cohort, excluding the predictors maternal and paternal cognitive ability.

	Cohort 1	Cohort 2		Cohort 3	
		Model 1	Model 2	Model 1	Model 2
SES	0.10**	0.30***	0.17***	0.28***	0.21***
HE - Child	-0.01	0.04	0.02	-0.02	-0.03
HE - Parents	0.04	0.06*	0.06*	0.07*	0.05*
Reading to the child	0.01	-	-	-	
PGS_{CA}	0.07	0.16***	0.14***	0.18***	0.13*
Openness	-	0.03	0.03	0.07**	0.06**
Age	0.18***	0.08***	0.09***	0.07**	0.06**
Sex	0.06	0.04	0.02	-0.06*	-0.07**
Birth weight	0.10***	0.11***	0.09***	-0.01	-0.01
Academic track	-	-	0.30***	-	0.27***
R^2	6.4%***	16.1%***	23.4%***	16.3%***	22.4%***

Note. **p* < .05, ***p* < .01, ****p* < .001

Table S5

Standardized regression estimates (β) of the multiple regression analyses for the outcome change in cognitive ability, separately for each cohort, excluding the predictors maternal and paternal cognitive ability.

	Cohort 1			Cohort 2			Cohort 3		
	Model 1	Model 2	Model 3	Model 1	Model 2	Model 3	Model 1	Model 2	Model 3
SES	0.28***	0.27***	0.20***	0.21***	0.10*	0.03	0.27***	0.10**	0.07*
HE - Child	-0.08	-0.07	-0.07	0.09*	0.06	0.05	-0.04	-0.02	-0.03
HE - Parents	0.01	-0.00	-0.01	0.08	0.00	0.01	0.06	0.04	0.04
Reading to the child	0.01	0.01	0.00	-	-	-	-	-	-
PGS_{CA}	0.09	0.07	0.05	0.13**	0.03	0.04	0.20***	0.12***	0.10**
Openness	0.11**	0.11**	0.10**	0.04	0.03	0.03	0.09*	0.03	0.03
Age	0.03	0.00	0.01	0.02	-0.03	-0.03	-0.01	-0.02	-0.02
Sex	-0.05	-0.05	-0.06	-0.02	-0.03	-0.04	-0.21***	-0.13***	-0.13***
Birth weight	0.09*	0.07	0.06	-0.00	-0.05	-0.06	-0.04	-0.00	-0.00
CA F2F 1	-	0.16***	0.13**	-	0.59***	0.54***	-	0.64***	0.61***
Academic track	-	-	0.17***	-	-	0.17***	-	-	0.09**
SOEP years of education	-	-	-	-	-	-	-	-	0.03
R^2	13.1%***	15.8%***	17.4%***	9.5%***	40.9%***	43.0%***	20.2%***	55.5%***	56.5%***

Note. **p < .05, **p < .01, ***p < .001*

Table S6

Explanatory standardized regression estimates (β) (and unstandardized total effects) of the decomposition analyses for the outcome cognitive ability at F2F 1 including academic track attendance, separately for each cohort (CA = cognitive ability, HE = home environment, F2F1 = first face-to-face interview).

	Cohort 2		Cohort 3	
	Direct effect	Indirect effect	Direct effect	Indirect effect
CA Mother	0.20***	0.10***	0.20***	0.09***
CA Father	0.21***	0.11***	0.24***	0.12***
HE - Child	0.01	0.00	-0.03	-0.00
HE - Parents	0.07**	0.00	0.06*	0.01*
PGS_{CA}	0.10**	0.02*	0.10*	0.02
Openness	0.02	0.00	0.06**	0.00*
Age	0.08***		0.07**	
Sex	0.03		-0.06**	
Birth weight	0.07***		-0.02	
Academic track	0.26***	0.11***	0.22***	0.08***
Direct effect of SES	-0.01		0.02	
Total indirect effect	0.31***		0.31***	
Total effect of SES	0.30***		0.33***	
R^2	30.0%***		31.0%***	

Note. **p < .05, **p < .01, ***p < .001*

Table S7

Explanatory standardized regression estimates (β) (and unstandardized total effects) of the decomposition analyses for the outcome change in cognitive ability including academic track attendance and years of education, separately for each cohort (CA = cognitive ability, HE = home environment, F2F1 = first face-to-face interview).

	Cohort 1		Cohort 2		Cohort 3	
	Direct effect	Indirect effect	Direct effect	Indirect effect	Direct effect	Indirect effect
CA F2F 1	0.11**	0.01*	0.49***	0.16***	0.60***	0.20***
CA Mother	0.11*	0.06*	0.13***	0.06***	0.03	0.01
CA Father	0.22***	0.11***	0.10*	0.05*	0.03	0.02
HE - Child	-0.06	-0.01	0.05	0.01	-0.02	-0.00
HE - Parents	0.02	0.00	0.01	0.00	0.04	0.01
Reading to the child	-0.02	-0.01	-	-	-	-
PGS_{CA}	0.04	0.01	0.02	0.00	0.10*	0.02*
Openness	0.10**	0.02*	0.03	0.00	0.03	0.00
Age	0.01		-0.03		-0.02	
Sex	-0.05		-0.04		-0.13***	
Birth weight	0.05		-0.07*		-0.00	
Academic track	0.14**	0.06**	0.16***	0.07***	0.09*	0.03*
SOEP years of education	-	-	-	-	0.03	0.01
Direct effect of SES	0.07		-0.03		0.05	
Total indirect effect	0.23***		0.37***		0.33***	
Total effect of SES	0.29***		0.33***		0.38***	
R^2	23.2%***		46.8%***		56.9%***	

Note. **p < .05, **p < .01, ***p < .001*

Table S8

Standardized regression estimates (β) of the multiple regression analyses for the outcome cognitive ability at F2F 1, separately for each cohort, including the predictors maternal and paternal PGS_{CA}.

	Cohort 1	Cohort 2		Cohort 3	
		Model 1	Model 2	Model 1	Model 2
SES	-0.02	0.08*	-0.00	0.07*	0.02
CA Mother	0.15***	0.26***	0.22***	0.22***	0.20***
CA Father	0.09*	0.21***	0.20***	0.25***	0.23***
HE - Child	-0.02	0.03	0.02	-0.02	-0.02
HE - Parents	0.07*	0.06*	0.06*	0.07**	0.06*
Reading to the child	-0.01	-	-	-	-
PGS_{CA} - Child	0.02	0.12*	0.11	0.16*	0.12
PGS_{CA} - Mother	-0.01	-0.04	-0.04	-0.00	0.01
PGS_{CA} - Father	0.09	-0.01	0.01	-0.02	-0.02
Openness	-	0.03	0.02	0.06**	0.06**
Age	0.18***	0.07**	0.08***	0.08**	0.07**
Sex	0.05	0.05*	0.04	-0.05*	-0.06**
Birth weight	0.10***	0.10***	0.08***	-0.02	-0.02
Academic track	-	-	0.26***	-	0.22***
R^2	10.4%***	24.8%***	29.9%***	27.2%***	31.2%***

Note. **p < .05, **p < .01, ***p < .001*; Cases for maternal and paternal PGS_{CA} between N = 427 and N = 836, depending on the cohort; Correlations across all cohorts: PGS_{CA}-Mother with SES (r = .18; p < .001), PGS_{CA}-Child (r = .50; p < .001), and CA Mother (r = .23; p < .001); PGS_{CA}-Father with SES (r = .15; p < .001), PGS_{CA}-Child (r = .49; p < .001), and CA Father (r = .19; p < .001).

Table S9

Standardized regression estimates (β) of the multiple regression analyses for the outcome cognitive ability at F2F 4, separately for each cohort, including the predictors maternal and paternal PGS_{CA}.

	Cohort 1			Cohort 2			Cohort 3		
	Model 1	Model 2	Model 3	Model 1	Model 2	Model 3	Model 1	Model 2	Model 3
SES	0.11	0.12*	0.07	0.06	0.03	-0.03	0.15**	0.06	0.05
CA Mother	0.15**	0.13**	0.13**	0.26***	0.15***	0.14***	0.15**	0.04	0.04
CA Father	0.25***	0.23****	0.22***	0.23***	0.10	0.10	0.14*	0.04	0.03
HE - Child	-0.07*	-0.07	-0.07	0.08*	0.06	0.05	-0.02	-0.01	-0.02
HE - Parents	0.03	0.02	0.02	0.06	0.00	0.01	0.05	0.04	0.04
Reading to the child	-0.01	-0.01	-0.02	-	-	-	-	-	-
PGS_{CA} - Child	0.11	0.10	0.09	0.14*	0.05	0.06	0.22***	0.15**	0.13*
PGS_{CA} - Mother	-0.07	-0.06	-0.06	-0.11	-0.03	-0.04	-0.02	-0.02	-0.01
PGS_{CA} - Father	-0.01	-0.02	-0.02	-0.02	-0.03	-0.02	-0.04	-0.02	-0.01
Openness	0.10**	0.11**	0.10**	0.04	0.03	0.03	0.09*	0.03	0.03
Age	0.03	0.01	0.01	0.01	-0.03	-0.03	-0.02	-0.02	-0.02
Sex	-0.04	-0.04	-0.05	-0.02	-0.04	-0.04	-0.19***	-0.12***	-0.13***
Birth weight	0.08*	0.06	0.05	-0.04	-0.06*	-0.07*	-0.05	-0.01	-0.01
CA F2F 1	-	0.12**	0.11**	-	0.53***	0.49***	-	0.61***	0.59***
Academic track	-	-	0.13**	-	-	0.16***	-	-	0.08*
SOEP years of education	-	-	-	-	-	-	-	-	0.03
R^2	21.6%***	22.8%***	23.5%***	23.4%***	45.4%***	46.9%***	25.8%***	56.6%***	57.3%***

Note. $*p < .05$, $**p < .01$, $***p < .001$; Cases for maternal and paternal PGS_{CA} between N = 427 and N = 836, depending on the cohort; Correlations across all cohorts: PGS_{CA}-Mother with SES (r = .18; p < .001), PGS_{CA}-Child (r = .50; p < .001), and CA Mother (r = .23; p < .001); PGS_{CA}-Father with SES (r = .15; p < .001), PGS_{CA}-Child (r = .49; p < .001), and CA Father (r = .19; p < .001).

Table S10

Standardized regression estimates (β) of the multiple regression analyses for the outcome change in cognitive ability, only cases with available data on CA F2F 4.

	Cohort 1 (N = 765)			Cohort 2 (N = 772)			Cohort 3 (N = 704)		
	Model 1	Model 2	Model 3	Model 1	Model 2	Model 3	Model 1	Model 2	Model 3
SES	0.11*	0.12*	0.08	0.04	0.02	-0.03	0.14**	0.07	0.05
CA Mother	0.14**	0.12**	0.12**	0.21***	0.13***	0.12***	0.12**	0.03	0.03
CA Father	0.22***	0.21***	0.19***	0.20***	0.08	0.08	0.13**	0.05	0.04
HE - Child	-0.08	-0.08*	-0.07*	0.07	0.06	0.05	-0.02	-0.02	-0.02
HE - Parents	0.03	0.03	0.02	0.06	0.01	0.01	0.05	0.05	0.04
Reading to the child	-0.01	-0.01	-0.02	-	-	-	-	-	-
PGS_{CA}	0.05	0.04	0.03	0.08	0.01	0.03	0.18**	0.14**	0.12**
Openness	0.11**	0.11**	0.10**	0.05	0.04	0.03	0.09*	0.03	0.03
Age	0.03	0.01	0.01	0.00	-0.04	-0.04	-0.02	-0.02	-0.02
Sex	-0.05	-0.05	-0.06	-0.03	-0.04	-0.05	-0.19***	-0.13***	-0.14***
Birth weight	0.07	0.06	0.05	-0.05	-0.07*	-0.08*	-0.05	-0.01	-0.01
CA F2F 1	-	0.13**	0.11**	-	0.52***	0.48***	-	0.53***	0.52***
Academic track	-	-	0.13**	-	-	0.17***	-	-	0.09*
SOEP years of education	-	-	-	-	-	-	-	-	0.03
R^2	17.3%***	18.9%***	20.3%***	15.8%***	38.7%***	40.9%***	22.4%***	45.3%***	46.1%***

Note. *$*p < .05$, $**p < .01$, $***p < .001$*

Table S11

Standardized regression estimates (β) (and unstandardized total effects) of the decomposition analyses for the outcome change in cognitive ability, only cases with available data on CA F2F 4.

	Cohort 1 (N = 765)		Cohort 2 (N = 772)		Cohort 3 (N = 704)	
	Direct effect	Indirect effect	Direct effect	Indirect effect	Direct effect	Indirect effect
CA F2F 1	0.11**	0.01	0.48***	0.09***	0.52***	0.16***
CA Mother	0.12**	0.05*	0.12***	0.04**	0.03	0.01
CA Father	0.19***	0.08**	0.08*	0.03	0.04	0.02
HE - Child	-0.07*	-0.01	0.05	0.01	-0.02	-0.00
HE - Parents	0.02	0.00	0.01	0.00	0.04	0.01
Reading to the child	-0.02	-0.01	-	-	-	-
PGS_{CA}	0.03	0.01	0.03	0.00	0.11**	0.02*
Openness	0.10**	0.02*	0.04	0.00	0.03	0.00
Academic track	0.13**		0.17***		0.09*	
SOEP years of education	-		-		0.02	
Direct effect of SES	0.08		-0.03		0.05	
Total indirect effect	0.15***		0.20***		0.23***	
Total effect of SES	0.23***		0.16**		0.29***	
R^2	20.3%***		40.8%***		46.1%***	

Note. **p* < .05, ***p* < .01, ****p* < .001

Table S12

Standardized regression estimates (β) of the multiple regression analyses for the outcome cognitive ability at F2F 4 (second cognitive ability measurement) using inverse probability weighting of selective panel dropout (CA = cognitive ability, HE = home environment, F2F1 = first face-to-face interview, F2F4 = fourth face-to-face interview).

	Cohort 1 (N = 1642) Model 3	Cohort 2 (N = 1748) Model 3	Cohort 3 (N = 1575) Model 3)
SES	0.08	-0.04	0.05
CA Mother	0.14**	0.14***	0.03
CA Father	0.21***	0.10*	0.03
HE - Child	-0.07	0.04	-0.03
HE - Parents	0.04	0.02	0.04
Reading to the child	-0.05	-	-
PGS_{CA}	0.04	0.02	0.11**
Openness	0.11**	0.04	0.02
Age	-0.01	-0.04	-0.02
Sex	-0.04	-0.05	-0.13***
Birth weight	0.05	-0.07*	-0.01
CA F2F 1	0.11**	0.48***	0.58***
Academic track	0.13**	0.15***	0.10**
SOEP years of education	-	-	0.02
R^2	25.1%***	44.9%***	55.8%***

Note. *$*p < .05$, $**p < .01$, $***p < .001$*

Table S13

Standardized regression estimates (β) (and unstandardized total effects) of the decomposition analyses for the outcome change in cognitive ability using inverse probability weighting of selective panel dropout (CA = cognitive ability, HE = home environment, F2F1 = first face-to-face interview, F2F4 = fourth face-to-face interview).

	Cohort 1 (N = 1642)		Cohort 2 (N = 1748)		Cohort 3 (N = 1575)	
	Direct effect	Indirect effect	Direct effect	Indirect effect	Direct effect	Indirect effect
CA F2F 1	0.11**	0.01*	0.48***	0.16***	0.58***	0.20***
CA Mother	0.14**	0.07*	0.14***	0.07***	0.03	0.01
CA Father	0.21***	0.10***	0.10*	0.05*	0.03	0.01
HE - Child	-0.07	-0.01	0.04	0.01	-0.03	-0.00
HE - Parents	0.04	0.00	0.02	0.00	0.04	0.01
Reading to the child	-0.05	-0.02	-	-	-	-
PGS_{CA}	0.04	0.01	0.02	0.00	0.11**	0.02*
Openness	0.11**	0.02*	0.04	0.00	0.02	0.00
Age	-0.01		-0.04		-0.02	
Sex	-0.04		-0.05		-0.13***	
Birth weight	0.05		-0.07*		-0.01	
Academic track	0.13**		0.16***		0.10**	
SOEP years of education	-	-	-	-	0.02	
Direct effect of SES	0.08		-0.04		0.05	
Total indirect effect	0.18***		0.30***		0.28***	
Total effect of SES	0.26***		0.26***		0.33***	
R^2	25.1%***		44.9%***		55.8%***	

Note. *$p < .05$, **$p < .01$, ***$p < .001$
