# Supplementary material for: Socioeconomic Differences in Cognitive Ability Across Childhood and Adolescence: An Investigation of Genetic, Individual, and Environmental Factors
Source: J Intell. 2026 Apr 10;14(4):63. doi: 10.3390/jintelligence14040063 (PMC13118255; doi:10.3390/jintelligence14040063)
Supplement: Supplementary file 1 [file jintelligence-14-00063-s001.zip › Supplementary Material_Code_24.03.2026.pdf]

Syntax 1. Multiple regression analysis, exemplary for cohort 1 and model 3, outcome change in cognitive ability.

```
data: file = data.dat;

variable: names = familyid personid cohort sex age_F2F1 birthweight zygosity CA_F2F1 CA_F2F4
                  openness homeenvironment homeenvironment_p SES income ISCED_m ISCED_f
                  ISEI_m ISEI_f EGP_m EGP_f CA_m CA_f reading PGSCA age_F2F4 academictrack
                  SOEP_F2F4;
          missing = all (-99);
          usevar = familyid CA_F2F4 SES CA_m CA_f homeenvironment homeenvironment_p
                  PGSCA reading age_F2F4 sex birthweight openness CA_F2F1 academictrack:
          cluster = familyid;
          auxiliary (m) = ISCED_m ISCED_f ISEI_f EGP_m;
          subpopulation is (cohort == 1);

analysis: type = complex;
          processors = 4;
          estimator = mlr;

model: CA_F2F4 on age_F2F4 sex birthweight;
       CA_F2F4 on SES CA_m CA_f homeenvironment homeenvironment_p
       PGSCA reading openness CA_F2F1 academic track;
       age_F2F4 sex birthweight SES CA_m CA_f homeenvironment
       homeenvironment_p PGSCA reading openness CA_F2F1 academic track;

output: sampstat STDYX STDY;
```

Syntax 2. Decomposition analysis, exemplary for cohort 3, outcome change in cognitive ability.

```
data: file = data.dat;


variable: names = familyid personid cohort sex age_F2F1 birthweight zygosity CA_F2F1 CA_F2F4
                  openness homeenvironment homeenvironment_p SES income ISCED_m ISCED_f
                  ISEI_m ISEI_f EGP_m EGP_f CA_m CA_f reading PGSCA age_F2F4 academictrack
                  SOEP_F2F4;
          missing = all (-99);
          usevar = familyid CA_F2F4 SES CA_m CA_f homeenvironment homeenvironment_p
                  PGSCA age_F2F4 sex birthweight openness CA_F2F1 academictrack SOEP_F2F4:
          cluster = familyid;
          auxiliary (m) = ISCED_m;
          subpopulation is (cohort == 3);

analysis: type = complex;
          processors = 4;
          estimator = mlr;

model: CA_F2F4 on age_F2F4 sex birthweight academictrack SOEP_F2F4;
       CA_F2F4 on CA_m (b1);
       CA_F2F4 on CA_f (b2);
       CA_F2F4 on homeenvironment (b3);
       CA_F2F4 on homeenvironment_p (b4);
       CA_F2F4 on PGSCA (b5);
       CA_F2F4 on openness (b6);
       CA_F2F4 on CA_F2F1 (b7);

       CA_m on SES (a1);
       CA_f on SES (a2);
       homeenvironment on SES (a3);
       homeenvironment_p on SES (a4);
       PGSCA on SES (a5);
       openness on SES (a6);
       CA_F2F1 on SES (a7);

       CA_F2F4 on SES (c);
       age_F2F4 sex birthweight CA_m CA_f PGSCA CA_F2F1 academictrack SOEP_F2F4
       homeenvironment homeenvironment_p openness;

      CA_m with CA_f;
```

CA_m with homeenvironment;
CA_m with homeenvironment_p;
CA_m with openness;
CA_m with PGSCA;
CA_m with CA_F2F1;
CA_m with academictrack;
CA_m with SOEP_F2F4;
CA_m with age_F2F4;
CA_m with sex;
CA_m with birthweight;
CA_f with PGSCA;
CA_f with homeenvironment;
CA_f with homeenvironment_p;
CA_f with openness;
CA_f with CA_F2F1;
CA_f with academictrack;
CA_f with SOEP_F2F4;
CA_f with age_F2F4;
CA_f with sex;
CA_f with birthweight;

PGSCA with CA_F2F1;
PGSCA with homeenvironment;
PGSCA with homeenvironment_p;
PGSCA with openness;
PGSCA with academictrack;
PGSCA with SOEP_F2F4;
PGSCA with age_F2F4;
PGSCA with sex;
PGSCA with birthweight;
CA_F2F1 with academictrack;
CA_F2F1 with SOEP_F2F4;
CA_F2F1 with age_F2F4;
CA_F2F1 with sex;
CA_F2F1 with birthweight;
CA_F2F1 with homeenvironment;
CA_F2F1 with homeenvironment_p;
CA_F2F1 with openness;
academictrack with SOEP_F2F4;
academictrack with age_F2F4;
academictrack with sex;

```
academictrack with birthweight;
academictrack with homeenvironment;
academictrack with homeenvironment_p;
academictrack with openness;

SOEP_F2F4 with age_F2F4;
SOEP_F2F4 with sex;
SOEP_F2F4 with birthweight;
SOEP_F2F4 with homeenvironment;
SOEP_F2F4 with homeenvironment_p;
SOEP_F2F4 with openness;

age_F2F4 with sex;
age_F2F4 with birthweight;
age_F2F4 with homeenvironment;
age_F2F4 with homeenvironment_p;
age_F2F4 with openness;

sex with birthweight;
sex with homeenvironment;
sex with homeenvironment_p;
sex with openness;

homeenvironment_p with homeenvironment;
homeenvironment_p with openness;
homeenvironment with openness;



Model constraint:
New(a1b1 a2b2 a3b3 a4b4 a5b5 a6b6 a7b7 totalind total);
a1b1 = a1*b1; !Indirect effect of X on Y via M
a2b2 = a2*b2;
a3b3 = a3*b3;
a4b4 = a4*b4;
a5b5 = a5*b5;
a6b6 = a6*b6;
a7b7 = a7*b7;

totalind = a1*b1 + a2*b2 + a3*b3 + a4*b4 + a5*b5 + a6*b6 + a7*b7; !Total indirect effect of SES
total = a1*b1 + a2*b2 + a3*b3 + a4*b4 + a5*b5 + a6*b6 + a7*b7 + c; !Total effect of SES
output: sampstat STDYX STDY;
```
